# Water-Mediated Electronic Modulation in Boron–Nitrogen Multi-resonance Thermally Activated Delayed Fluorescence Emitters

**DOI:** 10.1021/acs.jpclett.6c00346

**Published:** 2026-03-12

**Authors:** Chen-Yu Lin, Jing-Han Shi, Ya-Chen Lin, Pi-Tai Chou

**Affiliations:** ‡ Department of Chemistry, 33561 National Taiwan University, Taipei 10617, Taiwan; § Center for Emerging Material and Advanced Devices, National Taiwan University, Taipei 10617, Taiwan

## Abstract

Using the prototypical “multiple resonance” (MR) emitters CzBN and BCzBN (also known as DtBuCzB), we uncover a previously unrecognized yet crucial complexation process between B/N MR cores and water molecules that profoundly alters their ground- and excited-state photophysics and photochemistry. This discovery originated from an unexpected new blue-shifted band of BCzBN (360 nm absorption/375 nm emission), which appeared, alongside the parent 467/480 nm bands, when trace water was present in organic solvents, such as tetrahydrofuran (THF). The same phenomenon was subsequently observed for CzBN. Water titration experiments with CzBN in THF reveal a 1­(CzBN)/3­(H_2_O) stoichiometry complex with a binding constant of 10.65 ± 0.66 M^–3^. Quantum-chemical calculations further support that a linear relay water trimer engages the boron center through an O­(H_2_O) → B­(CzBN) Lewis acid–base interaction and forms H­(H_2_O)···N­(CzBN) hydrogen bonds, thereby perturbing the MR-core planarity. This interaction raises the LUMO energy while preserving the alternating HOMO/LUMO distribution, allowing both the parent CzBN (475 nm) and water complex (370 nm) emissions to exhibit thermally activated delayed fluorescence (TADF). Further fluorescence titration and time-resolved emission studies reaffirm a ground-state equilibrium between CzBN, the 3H_2_O–CzBN complex, and second-shell water-solvated 3H_2_O–CzBN. Upon excitation, expulsion of water from the boron center occurs in solvated 3H_2_O–CzBN, creating a branching pathway that competes with TADF and yields the characteristic 475 nm CzBN emission. Water complexation is also observed in other BCzBN derivatives with enhanced boron Lewis acidity but is absent in those with diminished acidity, indicating that the static interaction of O­(H_2_O) → B­(MR) with water is indispensable for forming water–B/N MR complexes. These results uncover water as a previously overlooked yet ubiquitous perturbing agent in B/N-type MR systems, opening new opportunities for understanding and exploiting their behavior under aqueous influence.

## Graphical abstract

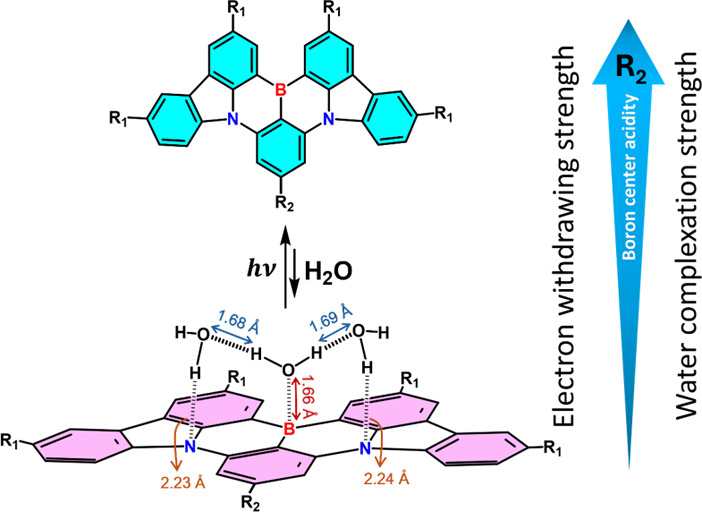

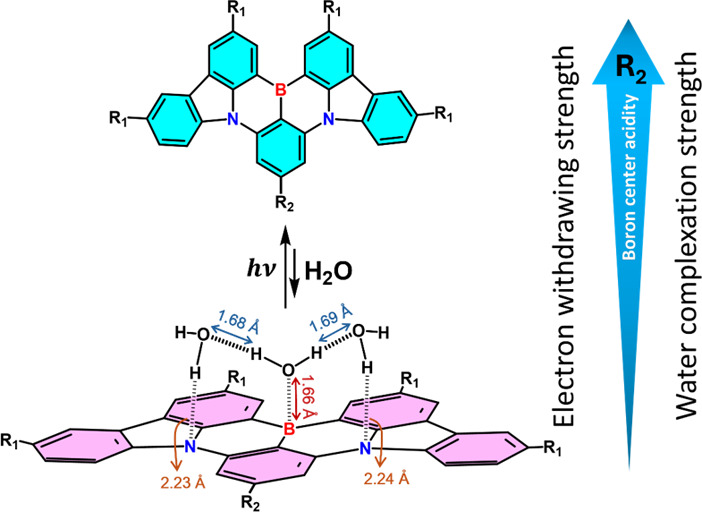

“Multiple resonance” (MR) compounds containing boron (B) and nitrogen (N) atoms have attracted considerable attention mainly because of their strong and narrowband emission.^1–6^ Moreover, the alternating distribution of the highest occupied molecular orbital (HOMO) and lowest unoccupied molecular orbital (LUMO) around the nitrogen and boron atoms, respectively, induces short-range charge transfer, which reduces the electron exchange/correlation energy and, hence, the energy gap (Δ*E*_S–T_) between lowest lying excited singlet (S_1_) and triplet (T_1_) states.^7^ In many designated B/N MR molecules, Δ*E*_S–T_ is small enough to promote the T_1_ → S_1_ reverse intersystem crossing, resulting in thermally activated delayed fluorescence (TADF) that is beneficial for harvesting triplet excitons in organic light-emitting diodes (OLEDs).^8^

From a chemistry point of view, B/N molecules possess a Lewis acid/base configuration within an alternating arrangement and, hence, are endowed with a unique chemical property. Recently, it has been reported that adding Lewis bases (e.g., molecules with lone pair electrons, anions, or carbenes) to BCzBN solution results in complex formation, leading to changes of absorption and emission (Scheme 1b).^9–11^ Such an interaction may significantly influence the intrinsic properties and, hence, applications, particularly in OLEDs. For example, many MR-TADF materials do not exhibit TADF in solution but only when dispersed in certain host matrices via vapor co-deposition; DABNA-1 is the representative example. Using time-resolved Fourier transform UV–vis absorption spectroscopy, we discovered a new transient excited state absorption induced by the interaction between 3,3′-di­(9*H*-carbazol-9-yl)-1,1′-biphenyl (mCBP, host) and DABNA-1 (guest; Scheme 1a), which gives rise to TADF. Conversely, the host–guest interaction is negligible in the bis­[2-(diphenylphosphino)­phenyl]­ether oxide (DPEPO, host)/DABNA-1 (guest) film, as evidenced by the absence of new transient absorption upon co-deposition.^12^ From a chemical structural perspective, the difference between mCBP and the DPEPO host lies in the presence of a basic nitrogen site in mCBP versus a neutral phosphine oxide group in DPEPO (Scheme 1a). It is reasonable to speculate that the nitrogen site of mCBP interacts with the boron site of DABNA-1.

**Scheme 1:**
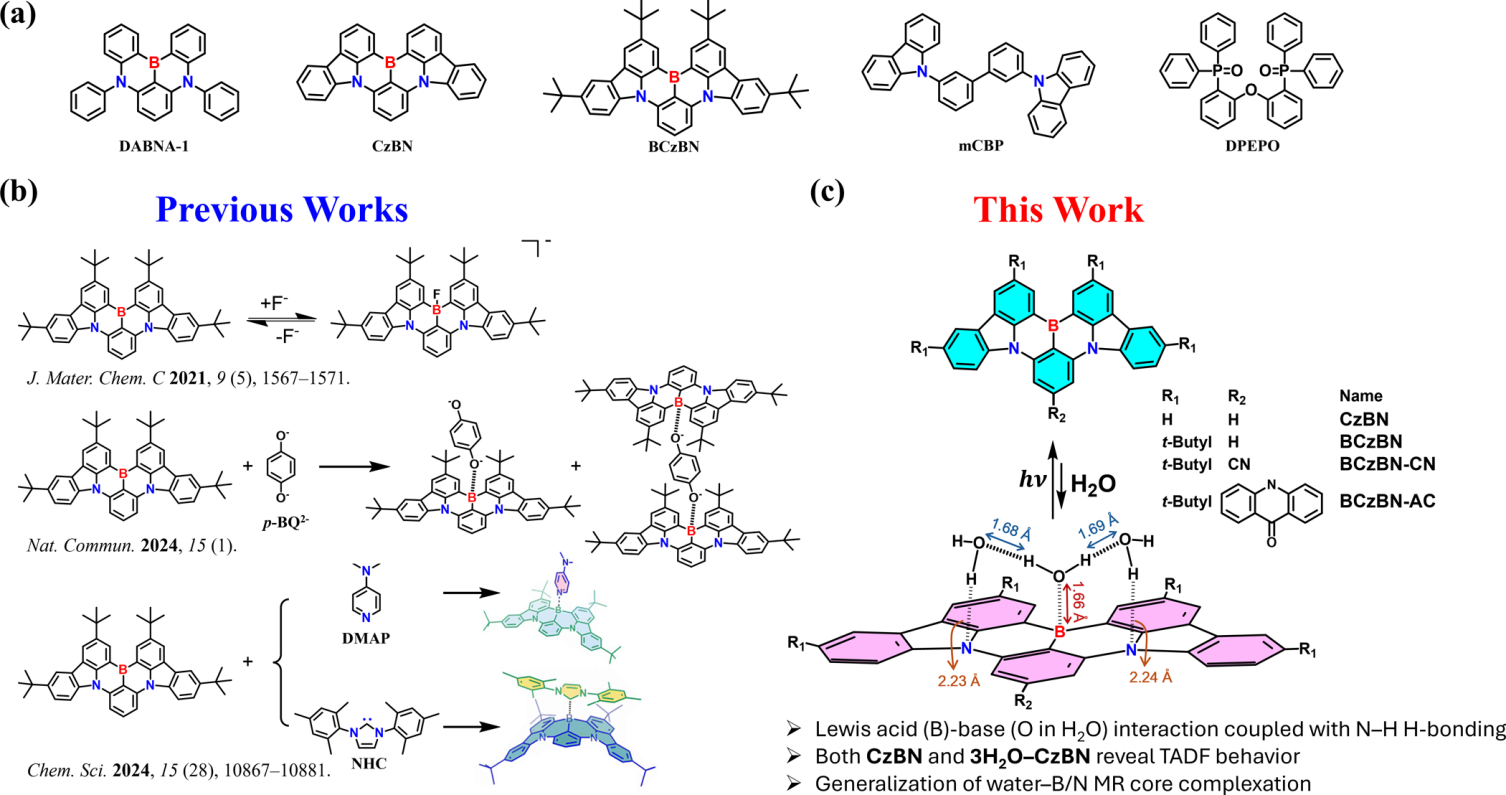
(a) Molecular Structure of the Compounds Discussed Throughout the Text: DABNA-1, CzBN, BCzBN, mCBP, and DPEPO, (b) Reaction Schemes Illustrating the Lewis Acid Behavior of MR Materials in Previous Works, (c) Proposed Reaction Scheme of Water–MR Core Complexation, Showing Various MR Molecules Examined in This Study^a^

a Key bond lengths are taken from the optimized 3H_2_O–CzBN structure obtained from theoretical calculations.

Building on B/N alternating Lewis acid/base interaction patterns, we report here the serendipitous discovery of complex formation between water molecules and B/N MR materials, exemplified (but not limited to) by the two prototype materials BCzBN and CzBN (Scheme 1a).^13–15^ The complexation of B/N materials with three water molecules significantly lifts the LUMO level without breaking the alternating HOMO/LUMO distribution, holding the MR effect with pronounced TADF properties. To the best of our knowledge, this is the first direct observation of a TADF process in a Lewis acid–base pair of MR materials under water perturbation. This water-promoted complexation mechanism can be extended to other B/N MR molecules, which is crucial for the future application of B/N MR molecules due to the ubiquity of water. The details of the results and discussion are elaborated below.

This research was initiated by serendipitous experimental observations. During studies of the solution-state photophysical properties of BCzBN, a well-known B/N-type MR-TADF compound, in various solvents, we observed an unexpected emission peak at 380 nm when the excitation wavelength was below 380 nm in some solvents [e.g., tetrahydrofuran (THF); Figure 1]. By contrast, the lowest lying absorption and emission peaks are known to be at 467 and 481 nm, respectively, for BCzBN in toluene.^14^ We therefore suspected that the 380 nm emission (with an excitation maximum near 350 nm) originated either from the aggregation effect or impurities.

**Figure 1:**
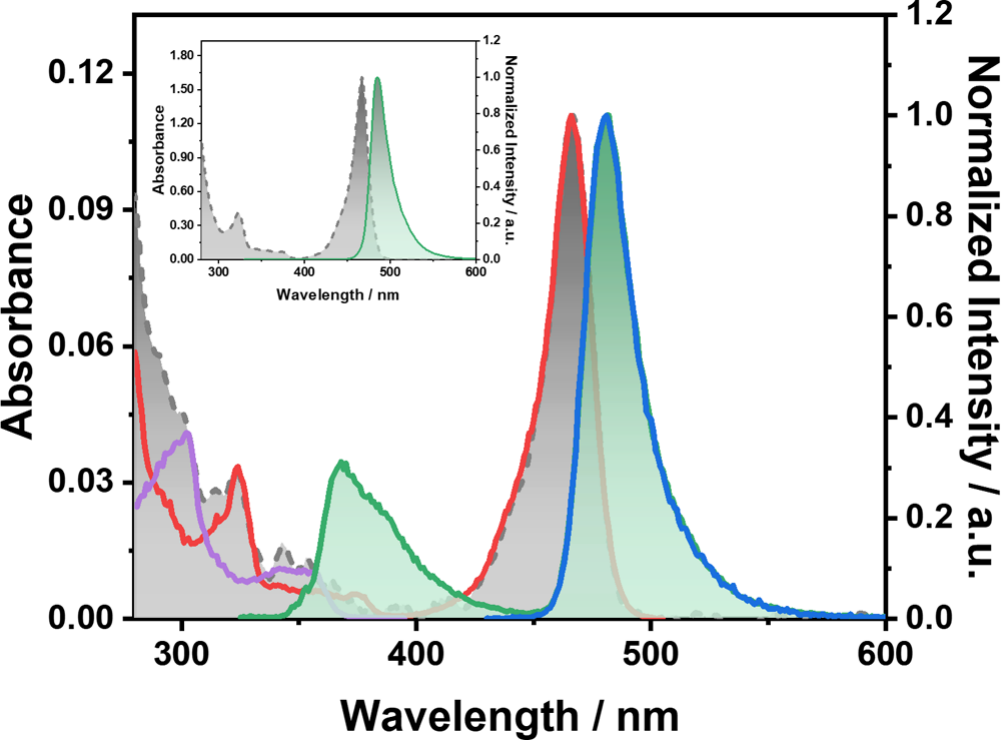
Steady-state absorption, emission, and excitation spectra of dilute BCzBN in unprocessed spectroscopic-grade THF, with the inset showing spectra in anhydrous THF. The absorption spectrum is depicted with a shaded, gray dashed line. The emission spectra upon excitation at 320 and 425 nm are displayed as green (shaded) and blue solid lines, respectively. The excitation spectra monitored at 510 and 400 nm are displayed as red and purple solid lines, respectively.

We first considered whether impurities in BCzBN caused this emission and found that, even after extensive purification by chromatography and recrystallization, with purity confirmed by NMR (Figure S4), the 380 nm emission peak persisted, ruling out impurities as the origin. Considering that vapor-deposited neat films of BCzBN have been reported to exhibit an excimer-like red-shifted emission,^16^ we then conducted concentration-dependent experiments on BCzBN in THF. As shown in Figure S8, the intensity ratio of the 380 nm emission decreased with an increasing BCzBN concentration, thus ruling out aggregation as the source of the 380 nm emission. Having eliminated all possible origins from BCzBN, we reasonably mulled over the purity of the solvent. Table S1 lists the spectroscopic-grade solvents used in this study. Using the same batch of BCzBN, the 380 nm emission was observed clearly in acetonitrile (ACN), diethyl acetate (EA), and THF but was not detectable in cyclohexane, toluene, or dichloromethane (Figure S9). The observation of 380 nm emission in these three different solvents has evidently excluded the skepticism of impurities from tetrahydrofuran or any specific solvent. A careful examination of the solvents listed in Table S1 reveals that ACN, THF, and EA all possess high water solubility, with ACN and THF being miscible with water.^17^ Conversely, water is sparsely soluble in cyclohexane and toluene and only slightly soluble in dichloromethane. This observation suggests that trace water could be responsible for the 380 nm emission. To test this hypothesis, THF was thoroughly dried by distillation over a drying agent, after which the 380 nm emission of BCzBN solution became negligible (see the inset of Figure 1).

A similar result, namely, the water-induced perturbation, was observed for CzBN (Figure S10). To investigate this behavior, we performed water-titration experiments in anhydrous THF. CzBN was selected instead of BCzBN for both absorption and fluorescence titration studies due to its better solubility in the water/THF mixture (*vide infra*). As shown in Figure 2a, the gradual addition of water to the CzBN solution led to the emergence of a new absorption band at 346 nm, accompanied by the disappearance of the original 457 nm peak. Correspondingly, the fluorescence spectra (Figure 2b) exhibited a progressive increase in the emission at 370 nm and a simultaneous decrease at 475 nm. Clear absorption isosbestic points appeared at 310, 324, and 360 nm, signifying a two-species equilibrium between monomeric CzBN (PLQY_areated_ = 83%) and a CzBN/H_2_O complex (PLQY_areated_ = 74%; see the Supporting Information) with a well-defined stoichiometry in the ground state. The titration plots of absorbance at 457 nm (*A*_457 nm_) and the emission intensity ratio (*F*_370 nm_/*F*_475 nm_) as a function of the water concentration are shown in Figure 2c and d, respectively. Fitting these plots with eqs S11 and S16 (see the Supporting Information) yielded consistent results (red lines in Figure 2c and d), indicating a stoichiometric coefficient of *m* ≈ 3, that is, the formation of a 3H_2_O–CzBN complex. The corresponding binding constant was determined to be 10.65 ± 0.66 M^–3^ in THF (Figure S23).

**Figure 2:**
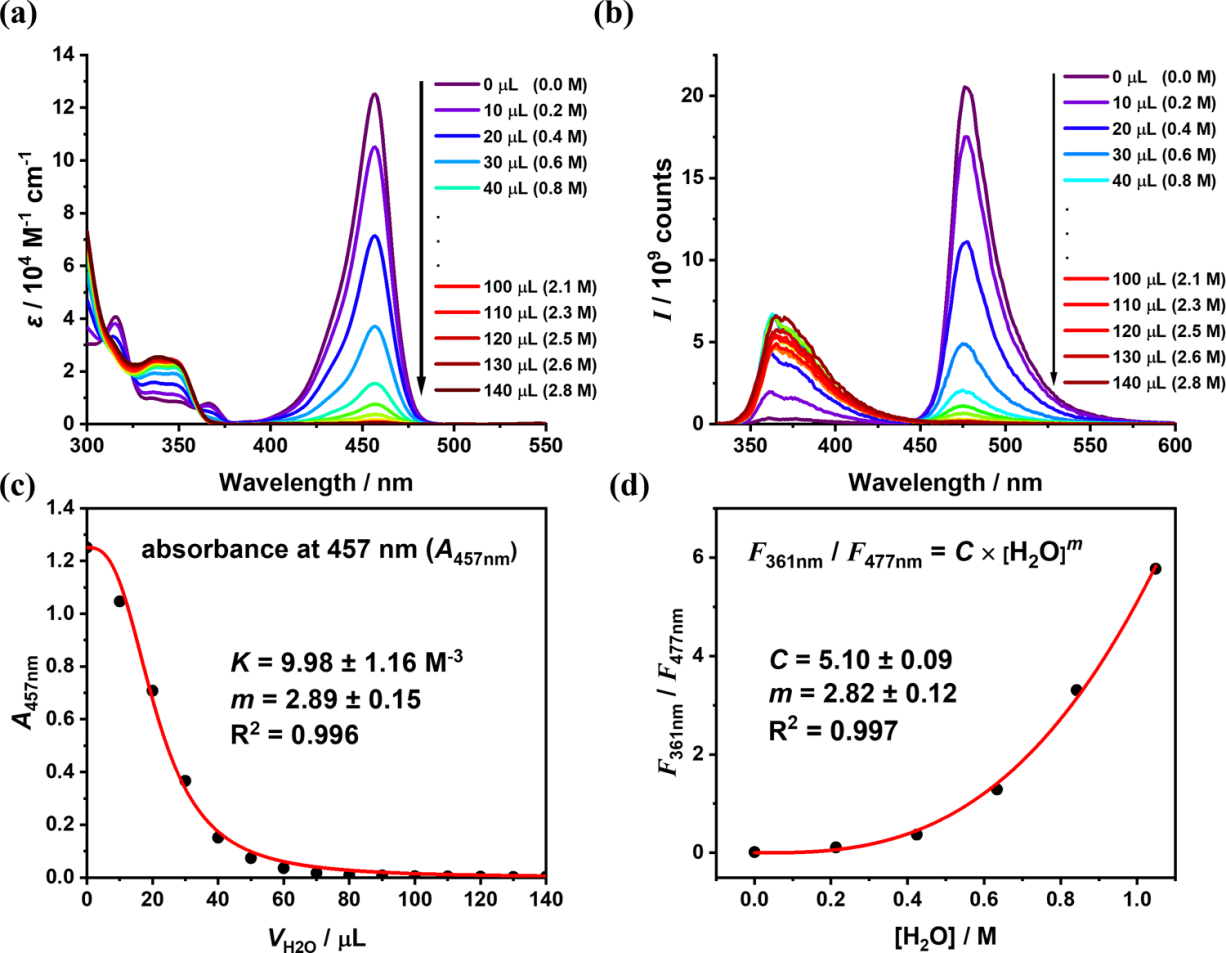
Spectral changes of CzBN in (a) absorption and (b) fluorescence spectra (λ_ex_ = 320 nm) upon gradual addition of water (0–140 μL, corresponding to 0–2.8 M) into THF solution (10^–5^ M, 2.6 mL). Both absorbance and photoluminescence intensity were calibrated to account for volume variations during titration, expressed as the extinction coefficient (ε) and molar PL intensity (*I*). The counts in the *y* axis have been calibrated by the dilution of the sample concentration due to the added water volume. (c) Fitting result of the titration plots of absorbance at 457 nm (*A*_457 nm_) versus volume of added water (*V*_H_2_O_) by using eq S11. (d) Fitting result of the titration plots of the PL intensity ratio (*F*_370 nm_/*F*_475 nm_) versus water concentration by using eq S16. Black dots represent the experimental data, and red lines represent the fitted curves. Both fitting results yield a stoichiometric coefficient of *m* = 3.

Notably, BCzBN also formed a water-incorporated complex in THF that displayed an additional emission band near 380 nm (Figure S9). However, due to the poor solubility of BCzBN upon hydration, likely arising from its bulky hydrophobic *tert*-butyl substituents, further titration analysis was not feasible (Figure S11). Consequently, all subsequent kinetic and spectroscopic studies were performed using CzBN as the representative system.

It is worth mentioning here that, in the presence of trace butylated hydroxytoluene (BHT), a common stabilizer in THF and many other organic solvents, the water-titration experiments exhibited significantly suppressed spectral changes (Figure S13). This behavior is attributed to the formation of a BHT–water core–shell structure, in which BHT serves as the hydrophobic core and the THF–water mixture forms the surrounding shell.^18^ Therefore, in THF containing the stabilizer BHT, virtually no free water molecules remain available to perturb CzBN. In yet another approach, though quantitative titration could not be performed in other solvents, such as ethyl acetate and acetonitrile, due to their limited water and compound solubility, steady-state absorption, emission, and time-resolved measurements consistently indicate similar water complex formation in these solvents (see Figures S9, S10, and S20, *vide infra*).

To validate the proposed 3H_2_O–CzBN complex, density functional theory (DFT) and time-dependent DFT (TD-DFT) calculations were carried out at the ωB97XD/6-31+G­(d,p) level in tetrahydrofuran [polarizable continuum model (PCM)] to examine the interaction between *n* water molecules and CzBN.^19–21^ The results show that systems containing one or two water molecules exhibit HOMO/LUMO characteristics and the lowest energy excitations around 370 nm (Table S4 and Figures S24–S26), comparable to those of pristine CzBN. Notice that several studies have shown that TD-DFT calculations fail to reproduce experimental lowest excitation energies (∼450 nm) and Δ*E*_S–T_ for MR emitters quantitatively (often overestimating them).^22–25^ Nevertheless, DFT and TD-DFT calculations still provide reliable qualitative information on molecular geometries and frontier orbitals; such insight and trends are sufficient for the aims of this study.

Moreover, coordination with three water molecules induces pronounced structural distortion, resulting in a less planar MR core (∑∠CBC = 341.96° vs 359.96° for pure CzBN) and a shortened B···O distance of 1.66 Å, indicative of electron donation from water to the boron center of the MR core (Figure 3b). Additionally, the remaining two H_2_O molecules engage in weak but significant hydrogen bonding with the nitrogen atoms at distances of 2.24 and 2.23 Å, respectively (see Scheme 1c). The synergistic contributions from the B···O Lewis acid–base interaction and N···H_2_O hydrogen bonding (H bonding) collectively stabilize the 3H_2_O–CzBN complex.

**Figure 3:**
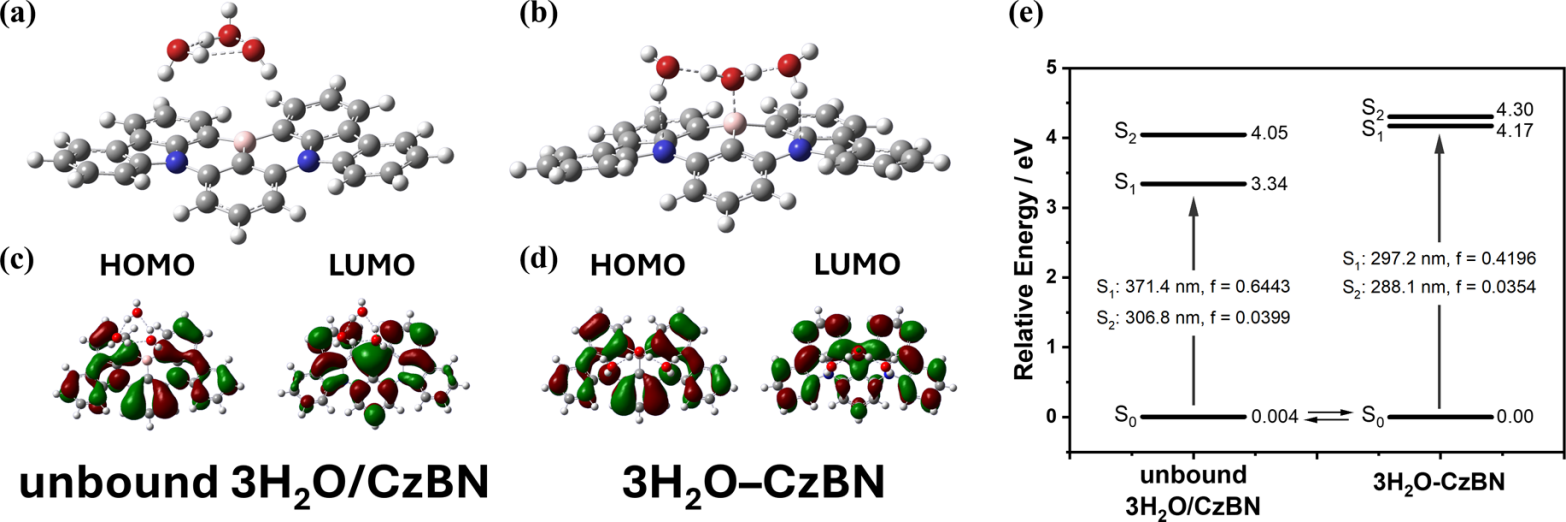
Optimized molecular structures and HOMO (left) and LUMO (right) distribution of (a and c) unbound 3H_2_O/CzBN and (b and d) 3H_2_O–CzBN complex. (e) Calculated energy diagram (eV) for unbound 3H_2_O/CzBN and 3H_2_O–CzBN complex.

The interaction between the oxygen and boron atoms elevates the LUMO energy level, thereby widening the HOMO–LUMO gap and producing a pronounced blue shift in both the absorption and emission spectra. The S_0_ → S_1_ transition of the 3H_2_O–CzBN complex was calculated to occur at 297 nm. Although this value overestimates the excitation energy relative to the experimentally observed 346 nm, it is nevertheless consistent with the experimentally detected blue shift during the water-titration experiments. For comparison, a configuration with the 3H_2_O cluster spatially separated from CzBN (denoted as unbound 3H_2_O/CzBN) was also optimized. The bound 3H_2_O–CzBN complex is calculated to possess a slightly lower energy than that of the unbound configuration by 0.004 eV (Figure 3e), agreeing with the experimentally observed weak binding constant of 10.65 M^–3^. Interestingly, electron donation to the boron center does not disrupt the alternating HOMO/LUMO distribution, a defining feature of the MR effect (Figure 3d), in contrast to the BCzBN/F^–^ system, where the strong B–F bond completely suppresses the MR character.^10^ Owing to the preservation of the MR framework, the 3H_2_O–CzBN complex is thus expected to retain TADF behavior (*vide infra*).

Based on multichannel scaling (MCS) measurements, Figure 4 presents the emission dynamics (λ_ex_ = 355 nm) of CzBN in THF containing 0.6 M water. Under steady-state conditions, an equilibrium is established between CzBN and its hydrated complex 3H_2_O–CzBN, leading to dual emissions at 370 and 475 nm (Figure 2). With monitoring at 380 nm attributed to the emission of 3H_2_O–CzBN, the kinetic trace clearly reveals triple-exponential decay components consisting of a prompt decay (<100 ns), an intermediate component (7.2 μs), and a long-lived component (67.9 μs). The prompt component was further resolved to 3.9 ns using a time-correlated single-photon counting (TCSPC) setup (Figure S14). In contrast, MCS measurements monitored at 500 nm, corresponding to the parent CzBN emission, display a kinetic profile comprising a prompt decay (<100 ns), a distinct rise component (4.2 μs), and a long-lived decay component (60.6 μs) (Figure 4a). Subsequent TCSPC measurements refine the prompt decay to 5.3 ns (Figure S14). Because excitation at 355 nm unavoidably excites both the unbound (water-free) CzBN monomer and the 3H_2_O–CzBN complex, the observation of prompt 500 nm emission with a 5.3 ns decay followed by TADF (60.6 μs) is reasonable.

**Figure 4:**
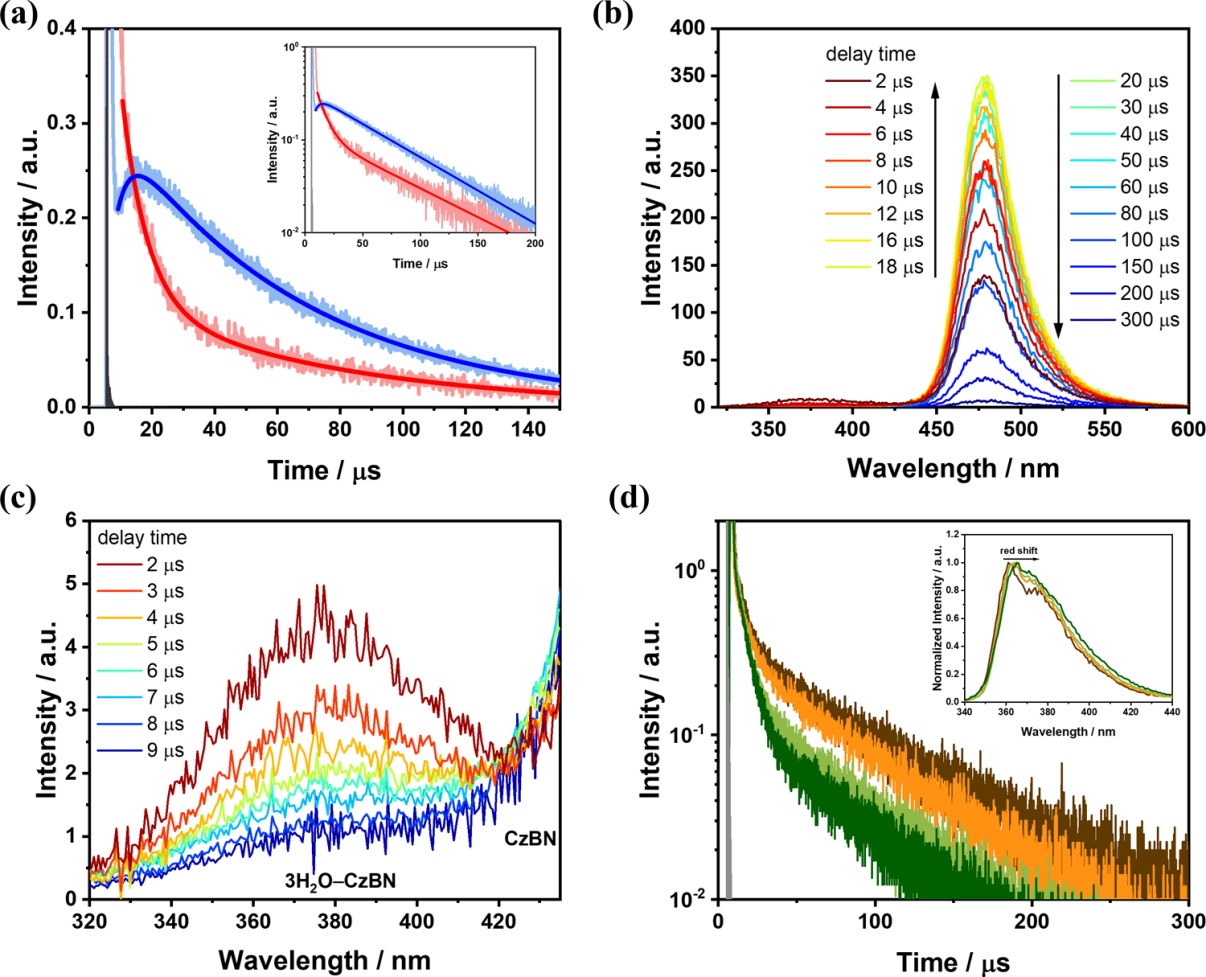
Time-resolved fluorescence profiles of CzBN (10^–5^ M, 2.6 mL) with 0.6 M water in degassed THF. (a) Kinetic traces monitored at 380 nm (red) and 500 (blue). (Inset) Identical data presented on the logarithmic intensity scale. (b) Time-resolved emission spectra covering both 370 and 475 nm bands, showing a distinct rise–decay behavior at 475 nm. (c) Enlarged view around 370 nm. (d) Water-concentration-dependent TADF dynamics over 0.2–2.1 M (red, 0.2 M; orange, 0.6 M; light green, 1.0 M; and dark green, 2.1 M) monitored at 380 nm. (Inset) Normalized PL spectra of CzBN upon the gradual addition of water (0.2–2.1 M) to a THF solution (CzBN, 10^–5^ M; λ_ex_, 320 nm). (a and d) Instrument response function (IRF) is depicted in the shaded, gray line for reference. For raw data and detailed information, see the Supporting Information.

For comparison, MCS measurements were also performed on water-free CzBN (in anhydrous THF) under identical excitation conditions. The kinetic trace at 500 nm exhibits a typical prompt decay (<100 ns) and TADF behavior but no discernible 4.2 μs rise component (Figure S15b). In addition, the 4.2 μs rise at 500 nm is on the same magnitude as the 7.2 μs decay component of the 380 nm emission band (Figure 4a), indicting a precursor–successor relationship. This correlation is further supported by the fitting results, in which fixing the population lifetime values (τ_2_) of both profiles during simulation still reproduces the experimental data satisfactorily (Figure S16). Taken together, these results support a mechanism in which, upon excitation, the 3H_2_O–CzBN complex not only undergoes radiative decay at 380 nm but also follows a branching pathway involving water expulsion, yielding CzBN* (* denotes the excited state), which subsequently emits at 475 nm.

Notably, while a previous study on the DMAP–BCzBN complex suggested a prompt S_1_ photodissociation pathway,^9^ our water-mediated system exhibits distinct behavior. The absence of free CzBN emission in the presence of 2.8 M water under aerated conditions (Figure 2b) effectively rules out the S_1_ photodissociation mechanism in the 3H_2_O–CzBN complex. The observed 4–7 μs long rise time constant suggests that water expulsion from 3H_2_O–CzBN most plausibly occurs within the triplet manifold. Fundamentally, the driving force for expulsion of water from the 3H_2_O–CzBN complex can be understood in terms of excited-state electron-density redistribution (Figure 3d). Upon the HOMO → LUMO transition, both the S_1_ and T_1_ states display a markedly increased electron density at the boron center. Photoexcitation therefore effectively reverses the Lewis acidity of the ground-state boron site, imparting a Lewis base-like character in the excited state. Consequently, the B–O interaction is significantly weakened, facilitating water release and the formation of emissive CzBN* species at 475 nm. Further evidence is provided by the photoluminescence (PL) intensity ratios of degassed versus aerated solutions. Upon excitation at both CzBN and 3H_2_O–CzBN absorption bands (e.g., λ_ex_ = 320 nm), the ratio (1.85) is higher than that obtained when exciting CzBN alone (e.g., λ_ex_ = 430 nm and ratio = 1.47) (Figure S17a, b, and d), indicating additional CzBN emission arising from water expulsion in the T_1_ state of 3H_2_O–CzBN. Moreover, time-resolved spectral evolution under 355 nm excitation (Figure 4b) reveals that the PL intensity of CzBN* (475 nm) gradually increases over the 2–18 μs delay window, accompanied by a concomitant decrease in the 3H_2_O–CzBN emission band at 370 nm (Figure 4c), further corroborating the photoinduced water-expulsion mechanism.

It is worth noting that the comparison between the steady-state (Figure 2b) and time-resolved (Figure 4b and c) spectra evidence a strikingly different intensity ratio between 370 and 475 nm emission bands for a comparable water content. This discrepancy mainly arises from fundamentally distinct emission origins captured in the two measurements. In the aerated steady-state measurements, triplet excitons are effectively quenched; thus, the observed signal is dominated by a prompt fluorescence from the S_1_ state. In contrast, the microsecond time-resolved emission spectra are governed by triplet-state kinetics. The photodissociation pathway of the 3H_2_O–CzBN complex acts as a competitive non-radiative channel, reducing its own delayed emission intensity while simultaneously generating an additional population of free CzBN in the T_1_ state. This population subsequently contributes to the enhanced delayed emission of free CzBN through TADF. Consequently, the intensity ratio of free CzBN to the 3H_2_O–CzBN complex is markedly higher in the delayed emission regime compared to the prompt fluorescence observed in the steady-state spectra.

The computational result also clearly shows that 3H_2_O–CzBN still holds MR properties, i.e., the well-separated electron density between HOMO and LUMO (Figure 3d), inferring that 3H_2_O–CzBN possesses a small Δ*E*_S–T_, evidenced by the observed long-lived emission component monitored at 370 nm (see Figure 4a), which is quenched by oxygen and ascribed to the delayed fluorescence originating from TADF. Accordingly, two qualitatively distinct forms of the 3H_2_O–CzBN complex are identified, both exhibiting ∼370 nm delayed fluorescence but with different TADF time constants of 4–7 and ∼68 μs, respectively. We propose that the shorter lived TADF corresponds to 3H_2_O–CzBN species surrounded by additional solvating water molecules, in which photoinduced water expulsion can occur and subsequently produce CzBN* with a 475 nm emission. In contrast, the long-lived component is attributed to 3H_2_O–CzBN species that are essentially free from external water solvation, thereby sustaining a long-lived TADF.

Support for the above proposal is given by several additional experimental observations. First, the 370 nm emission band becomes slightly red-shifted and broadened as the water concentration increases (Figure S12 and inset of Figure 4d), consistent with solvatochromic behavior arising from water solvation of the emitting state. In such second-shell-like water environments, the water trimer released upon photoinduced 3H_2_O–CzBN dissociation can be further stabilized through additional hydrogen bonding or incorporation into larger, lower energy water clusters.^26^ Collectively, the presence of the second solvation shell lowers the internal energy of the photodissociation product, namely, (3H_2_O)_sol_, providing a thermodynamic impetus for the dissociation pathway; therefore, a concomitantly lower activation energy and an accelerated rate are expected. Coupled with the Lewis base-like character of the boron center in the excited state (Figure 3d), which weakens the B–O interaction, this solvation-enhanced stabilization facilitates an excited-state water-expulsion channel with a time constant of 4–7 μs. In contrast, at low water concentrations, where the 3H_2_O–CzBN complex is largely free of external water solvation, the absence of second-shell stabilization renders water expulsion relatively unfavorable. As a result, reverse intersystem crossing (RISC) dominates, giving rise to the long-lived 68 μs delayed component.

This mechanistic picture is further corroborated by kinetic analyses of CzBN in THF across varying water concentrations (Figure 4d). The pre-exponential factor of 370 nm emission decay associated with τ_2_ (4–7 μs) systematically increases with a higher water content (Table S3), supporting its assignment to water expulsion from second-shell-solvated 3H_2_O–CzBN species. This pathway competes kinetically with RISC via a more rapid decay channel. Assuming comparable intrinsic TADF dynamics between the solvated 3H_2_O–CzBN species and the unsolvated 3H_2_O–CzBN complex, the latter exhibits a longer TADF lifetime (68 μs).

With all of the results brought together, a unified mechanistic picture for the interplay between water and CzBN is proposed in Figure 5. In the ground state, CzBN (or BCzBN) exists in equilibrium with the 3H_2_O–CzBN complex and its second-shell-water solvated counterpart, denoted as (3H_2_O–CzBN)_sol_ in Figure 5. In the excited state, photoinduced expulsion of water from the boron center occurs exclusively in (3H_2_O–CzBN)_sol_, generating a branching deactivation pathway that competes with TADF and yields the characteristic CzBN emission at 475 nm. In contrast, the isolated 3H_2_O–CzBN complex lacks this dissociation channel and, therefore, exhibits 370 nm emission with long-lived TADF.

**Figure 5:**
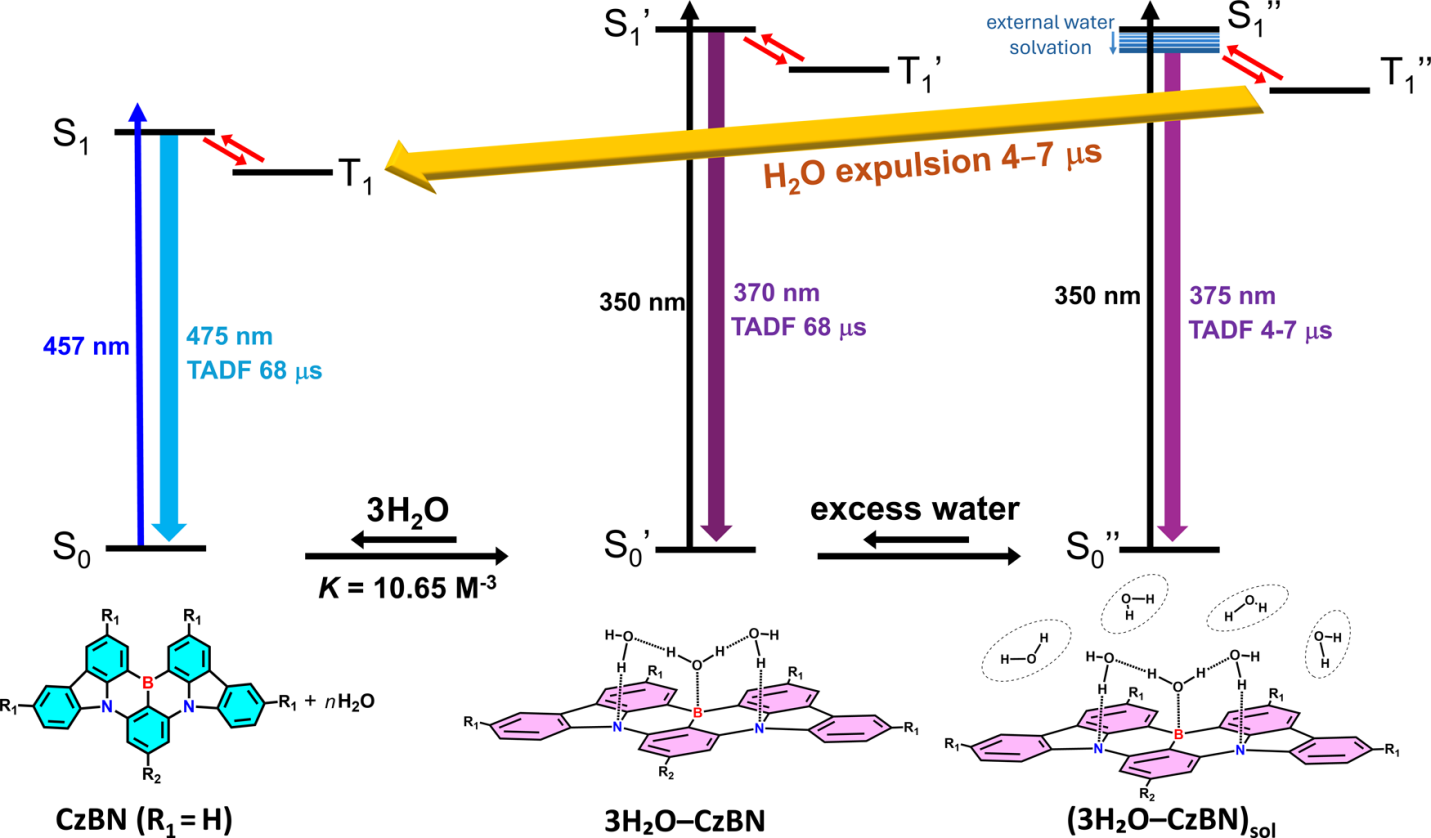
Proposed ground-state equilibrium among CzBN derivatives, 3H_2_O–CzBN, and water-surrounded (3H_2_O–CzBNs)_sol_ in THF. Notice that the surrounding water alters only the excited-state behavior, inducing a slight red shift in emission and promoting significant water expulsion. The absorption wavelengths, emission wavelengths, and lifetimes are represented by CzBN, 3H_2_O–CzBN, and (3H_2_O–CzBN)_sol_.

Beyond CzBN and BCzBN, we further examined water complexation in related B/N-type MR emitters. Since BCzBN and its derivatives are widely used in lighting applications,^1–6,27–34^ our investigation focused on analogues bearing different substituents at the R_2_ position (Scheme 1c), to modulate the boron Lewis acidity via resonance or inductive effects. To this end, BCzBN–CN and BCzBN–AC (Scheme 1c) were synthesized (see the Supporting Information for synthetic details), and their emission titration behaviors toward water were measured (Figure S21). Strikingly, BCzBN–CN exhibited clear signatures of the water complex formation, namely, attenuation of the characteristic MR emission and concomitant growth of a greatly blue-shifted emission band, whereas BCzBN–AC displayed no observable response. This contrasting behavior is readily rationalized by the increased Lewis acidity of the boron center induced by the electron-withdrawing −CN group, in sharp contrast to the decrease in boron Lewis acidity due to the electron-donating substituent in BCzBN–AC. Collectively, a simplified yet generalizable B/N unit capable of water complexation is illustrated in Figure 6. We propose that B/N-type MR compounds incorporating a core motif similar to that shown in Figure 6 possess intrinsic potential for binding water, where the O­(H_2_O) → B­(MR) Lewis acid–base interaction plays a central role in stabilizing the resulting water–B/N MR complexes. Further functionalization that enhances the boron acidity, such as introducing more strongly electron-withdrawing substituents on the −R group in Figure 6, should correspondingly increase the water association constant. These findings open new opportunities for investigating the photophysics and photochemistry of B/N-based MR materials under ubiquitous aqueous perturbations.

**Figure 6:**
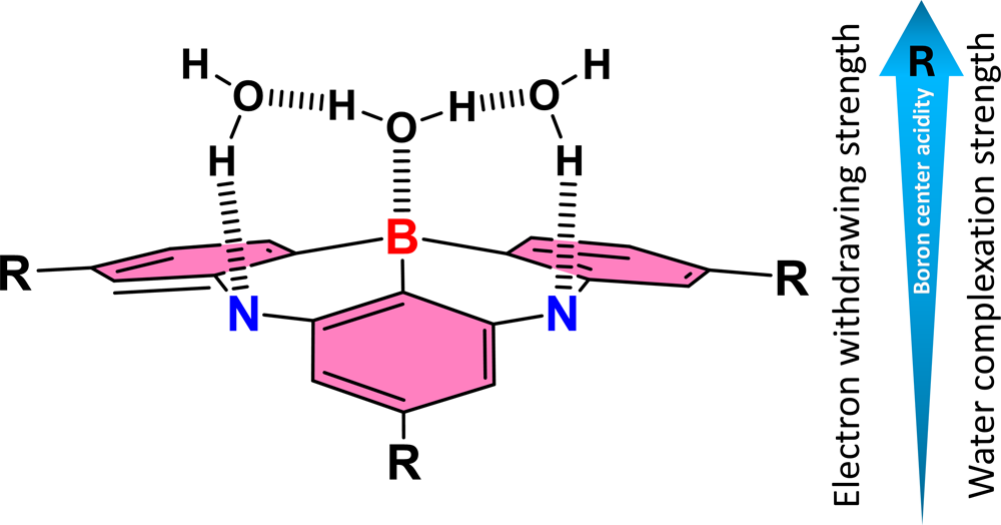
Prototypical B/N core capable of undergoing water complexation can be envisioned in which the B and two N atoms occupy *ortho* positions on two adjacent benzene rings, while these two N atoms reside in *meta* positions on a third benzene unit. This spatial arrangement creates a triangular coordination pocket that is ideally suited to hosting a relay of three water molecules.

However, we note that the present work focuses on the fundamental mechanism of water–CzBN complexation mediated by Lewis acid–base and hydrogen-bonding interactions as well as the associated water expulsion process in the excited state. The relatively high water concentrations required to observe this effect, together with stringent moisture-control practices during the fabrication of the OLED, are expected to limit the extent of water interference in practical B/N-type MR OLED devices. Nevertheless, the observations and mechanistic insights presented here elucidate the broader role of water in modulating the photophysics of MR-TADF systems.

In summary, we have identified a previously unrecognized complexation reaction between prototypical B/N MR emitters, such as CzBN and water molecules. Fluorescence titration of water in THF reveals a ground-state equilibrium involving free CzBN, the 3H_2_O–CzBN complex, and its second-shell-solvated counterpart (3H_2_O–CzBN)_sol_, Theoretical calculations corroborate the 3:1 structural stoichiometry, featuring a central H_2_O molecule engaging the boron center through a short B–O interaction and two peripheral H_2_O···N­(CzBN) hydrogen bonds. In the excited state, both 3H_2_O–CzBN and (3H_2_O–CzBN)_sol_ display TADF at ∼370–375 nm. However, only (3H_2_O–CzBN)_sol_ undergoes an additional excited-state water-expulsion pathway with a rate constant of (4–7 μs)^–1^, producing the 475 nm CzBN TADF emission. The overall mechanistic framework is summarized in Figure 5. This water-mediated B/N MR configuration appears to generalize to other B/N-type MR systems, particularly among BCzBN analogues, thereby opening a new avenue for exploring MR materials under aqueous perturbation.

## Supplementary Material



## Notes

Details of synthetic procedures, ^1^H NMR spectra, additional experimental details, photophysical measurements, details of binding constant fitting, and computational approach (PDF)
