# Supplementary material for: Water-Mediated Electronic Modulation in Boron–Nitrogen Multi-resonance Thermally Activated Delayed Fluorescence Emitters
Source: J Phys Chem Lett. 2026 Mar 12;17(12):3530–8. doi: 10.1021/acs.jpclett.6c00346 (PMC13034463; doi:10.1021/acs.jpclett.6c00346)
Supplement: Supplementary file 1 [file jz6c00346_si_001.pdf]

Supporting Information

Water-Mediated Electronic Modulation in Boron–Nitrogen Multi-Resonance TADF Emitters

Chen-Yu Lin[1+], Jing-Han Shi[1+], Ya-Chen Lin[1], Pi-Tai Chou[1,2] *

Pi-Tai Chou, email: chop@ntu.edu.tw

[1] Department of Chemistry, National Taiwan University, Taipei, 10617, Taiwan.

[2] Center for Emerging Material and Advanced Devices, National Taiwan University, Taipei 10617, Taiwan

[+] *equal contribution*

Table of Contents

1. **General Experimental Section** **S-2**
2. **Synthesis and Characterization of Compounds** **S-2**
3. **Photophysical Properties** **S-12**
   Steady-State Spectra S-12
   Lifetime Measurements S-12
   Time-resolved Photoluminescence Spectra S-13
   Fitting of binding constant (K): S-21
4. **Computational Section** **S-24**
5. **References** **S-29**

1. General Experimental Section

Chemicals and solvents were purchased from Sigma-Aldrich, Acros Organics, Combi-Blocks, AK scientific. All commercial reagents were used without purification unless mentioned otherwise. Anhydrous solvents were either obtained by distillation under dry nitrogen gas after drying with CaH_2 or directly purchased as anhydrous. The thin-layer chromatography (TLC) was performed on precoated Merck silica gel 60 F254 alumina plates (0.25 mm), and the spots were visualized by UV lamps (254 nm / 365 nm). Purification with column chromatography was carried out using Geduran® silica gel 60 (0.040-0.063 mm) from Merck. ^{1}H and ^{13}C NMR spectra were recorded on a Bruker AVIII HD 400 spectrometer in $CDCl_3$. Chemical shifts (δ) were reported in parts per million (ppm) and calibrated using the solvent as the internal standard for ^{1}H ($CHCl_3$: 7.26 ppm) and ^{13}C NMR ($CDCl_3$: 77.16 ppm). Coupling constants (*J*) were reported in hertz (Hz), and the splitting patterns were reported as s (singlet), d (doublet), dd (doublet of doublet), t (triplet).

2. Synthesis and Characterization of Compounds

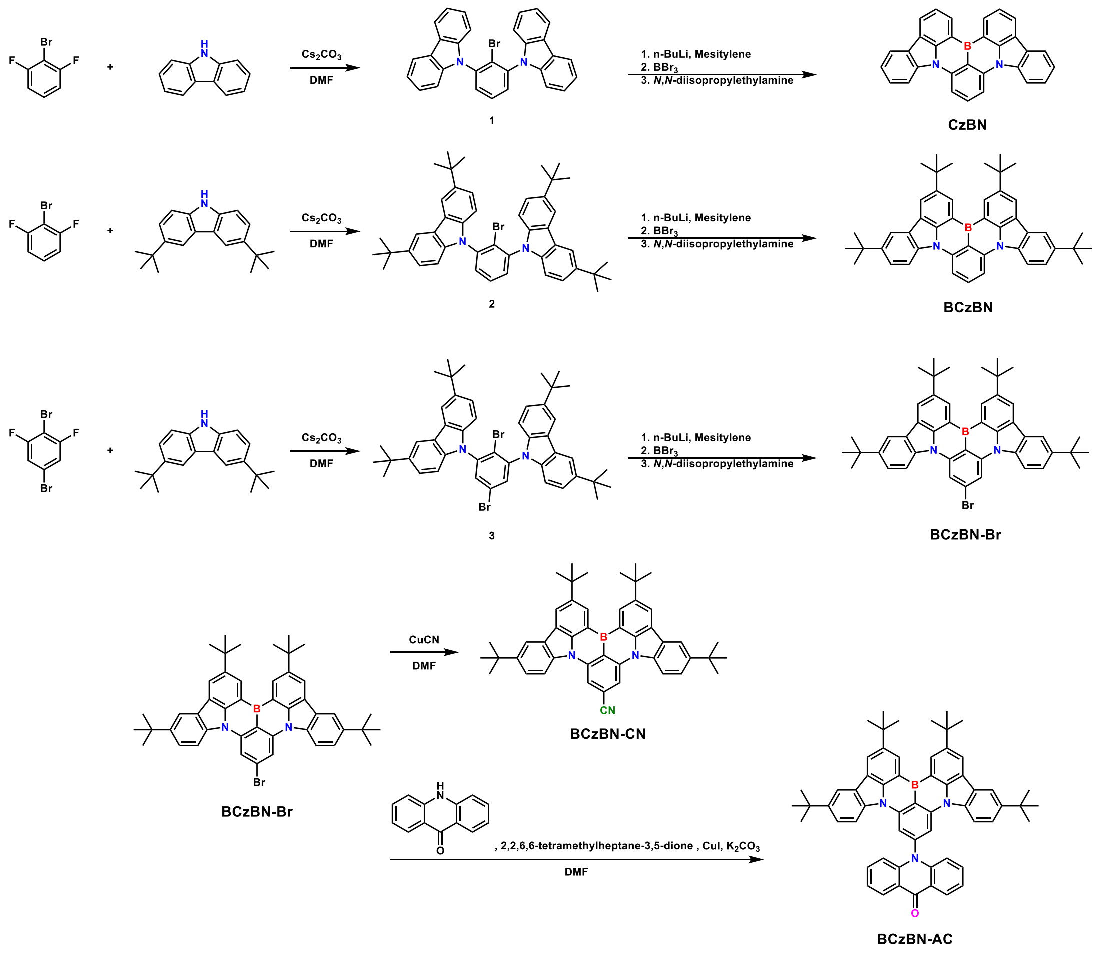

9,9'-(2-bromo-1,3-phenylene)bis(9*H*-carbazole) (1)

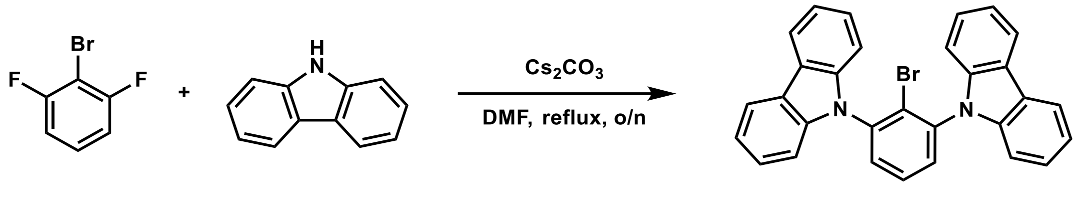

1

9*H*-carbazole (3.8 g, 23.1 mmol) was dissolved in 20 mL anhydrous *N*,*N*-dimethylformamide (DMF), and the solution was slowly added dropwise into an oven-dried 100 mL two-neck round-bottom flask with the mixture of cesium carbonate (Cs_2CO_3, 13.6 g, 42.0 mmol) and 10 mL anhydrous DMF over a period of 5 minutes. The suspension was stirred at 60 °C for 30 minutes, and 2-bromo-1,3-difluorobenzene (2.0 g, 10.5 mmol) was injected dropwise into it within 5 minutes. The solution was stirred under reflux overnight. The reaction mixture was cooled to room temperature, then poured into ice water. The mixture was filtered, and the solid was washed with water. The crude product was recrystallized with dichloromethane (DCM) and hexane (Hex) to afford compound **1** as a white solid (4.9 g, 95%).

^{1}H NMR ($CDCl_3$, 400 MHz): The spectroscopic data are in accordance to reported literature;[1] δ 8.18 (d, *J* = 7.7 Hz, 4H), 7.76 (dd, *J* = 9.2, 6.1 Hz, 1H), 7.73 – 7.68 (m, 2H), 7.48 (t, *J* = 7.6 Hz, 4H), 7.34 (t, *J* = 7.4 Hz, 4H), 7.21 (d, *J* = 8.1 Hz, 4H).

2,6-bis(9*H*-carbazol-9-yl) boron (CzBN)

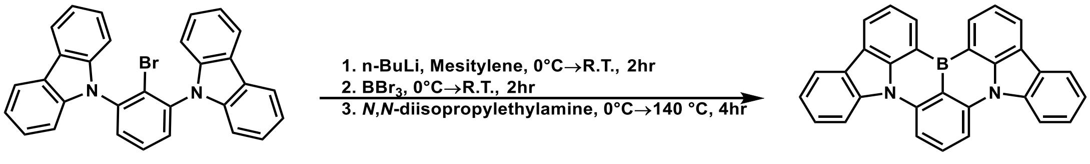

1 **CzBN**

Into an oven-dried 100 mL two-neck round-bottom flask was added compound **1** (2.4 g, 5.0 mmol) and anhydrous Xylene (30 mL) under N_2 atmosphere. The reaction mixture was cooled to 0 °C for 10 minutes, *n*-butyllithium (*n*-BuLi) in hexane (3.75 mL, 1.6 M, 6.0 mmol) was added dropwise, then stirring at room temperature for 2 hours. Boron tribromide (BBr_3, 0.7 mL, 7.0 mmol) was slowly added to the solution at 0 °C. After returning to room temperature, the mixture was stirred for 2 hours. The suspension was cooled to 0 °C for 10 minutes, and *N*,*N*-Diisopropylethylamine (DIPEA) (1.7 mL, 10.0 mmol) was added dropwise. After stirring for 10 minutes, the reaction was warmed to 140 °C for 4 hours. Then the mixture was cooled to room temperature, quenched with water, and extracted with DCM. The organic layer was dried over

Na_2SO_4 and filtered, and the solvent was removed under reduced pressure. The brown crude product was purified by column chromatography (silica gel, 20% DCM/Hex) to afford **CzBN** as a light-yellow solid (624.2 mg, 30%).

^{1}H NMR ($CDCl_3$, 400 MHz): The spectroscopic data are in accordance to reported literature;[1] δ 8.95 (d, *J* = 7.5 Hz, 2H), 8.47 (d, *J* = 8.4 Hz, 2H), 8.35 (t, *J* = 7.5 Hz, 4H), 8.23 (d, *J* = 7.6 Hz, 2H), 8.00 (t, *J* = 8.2 Hz, 1H), 7.67 (t, *J* = 7.4 Hz, 2H), 7.62 (t, *J* = 7.8 Hz, 2H), 7.45 (t, *J* = 7.3 Hz, 2H).

9,9'-(2-bromo-1,3-phenylene)bis(3,6-di-*tert*-butyl-9*H*-carbazole) (2)

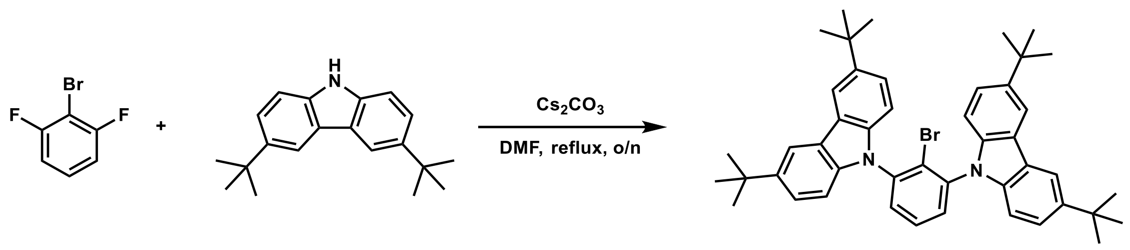

2

3,6-*tert*-butylcarbazole (4.8 g, 17.5 mmol) was dissolved in 20 mL anhydrous DMF. The solution was slowly added dropwise into an oven-dried 100 mL two-neck round-bottom flask with the mixture of Cs_2CO_3 (10.3 g, 31.8 mmol) and 10 mL anhydrous DMF over a period of 5 minutes. The suspension was stirred at 60 °C for 30 minutes, and 2-bromo-1,3-difluorobenzene (1.5 g, 7.9 mmol) was injected dropwise into it within 5 minutes. The solution was stirred under reflux overnight. After completion (determined by TLC monitor), the reaction mixture was cooled to room temperature, then poured into ice water. The mixture was filtered, and the solid was washed with water. The crude product was recrystallized with DCM and Hex to afford compound **2** as a white solid (3.7 g, 65%).

^{1}H NMR ($CDCl_3$, 400 MHz): The spectroscopic data are in accordance to reported literature;[2] δ 8.16 (d, *J* = 1.9 Hz, 4H), 7.71 – 7.65 (m, 1H), 7.65 – 7.59 (m, 2H), 7.51 (dd, *J* = 8.6, 1.9 Hz, 4H), 7.12 (d, *J* = 8.6 Hz, 4H), 1.48 (s, 36H).

BCzBN

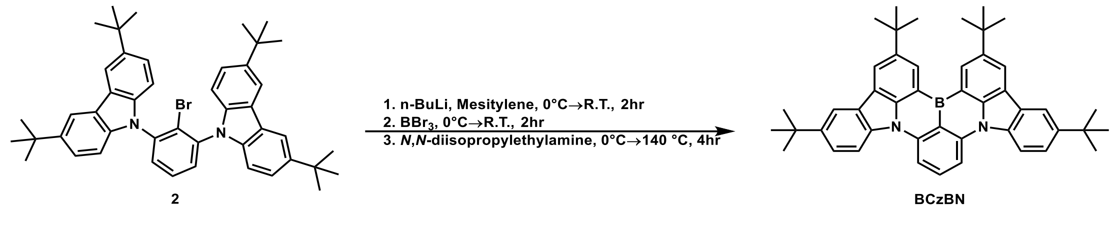

Into an oven-dried 100 mL two-neck round-bottom flask was added compound **2**

(2.3 g, 3.2 mmol) and anhydrous Xylene (30 mL) under N_2 atmosphere. The rection mixture was cooled to 0 °C for 10 minutes, *n*-BuLi in hexanes (2.37 mL, 1.6 M, 3.8 mmol) was added dropwise, then stirring at room temperature for 2 hours. BBr_3 (0.4 mL, 4.4 mmol) was slowly added to the solution at 0 °C. After returning to room temperature, the mixture was stirred for 2 hours. The suspension was cooled to 0 °C for 10 minutes, and DIPEA (1.1 mL, 6.3 mmol) was added dropwise. After stirring for 10 minutes, the reaction was warmed to 140 °C for 4 hours. Then the mixture was cooled to room temperature, quenched with water, and extracted with DCM. The organic layer was dried over Na_2SO_4 and filtered, and the solvent was removed under reduced pressure. The brown crude product was purified by column chromatography (silica gel, 20% DCM/Hex) to afford **BCzBN** as a light-yellow solid (451.1 mg, 22%).

^{1}H NMR ($CDCl_3$, 400 MHz): The spectroscopic data are in accordance to reported literature;[2] δ 9.13 (d, J = 1.9 Hz, 2H), 8.47 (d, J = 1.8 Hz, 2H), 8.39 (d, J = 8.8 Hz, 2H), 8.31 (d, J = 8.3 Hz, 2H), 8.27 (d, J = 2.0 Hz, 2H), 7.99 (t, J = 8.3 Hz, 1H), 7.66 (dd, J = 8.8, 2.1 Hz, 2H), 1.68 (s, 18H), 1.54 (s, 18H).

9,9'-(2,5-dibromo-1,3-phenylene)bis(3,6-di-*tert*-butyl-9*H*-carbazole) (3)

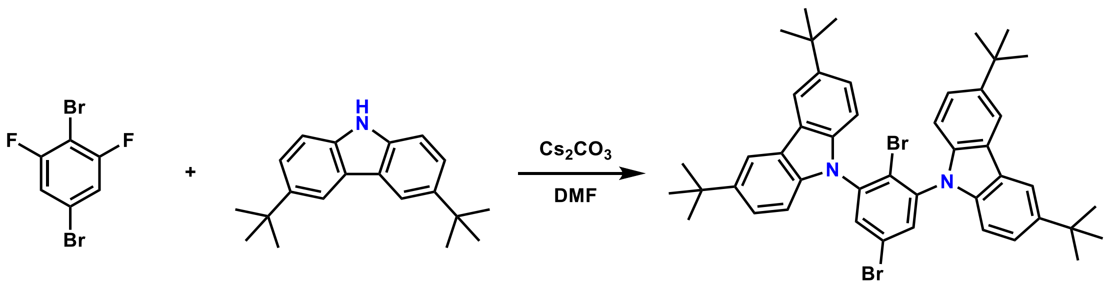

3

3,6-*tert*-butylcarbazole (6.1 g, 22 mmol) was dissolved in 20 mL anhydrous DMF. The solution was slowly added dropwise into an oven-dried 100 mL two-neck round-bottom flask with the mixture of Cs_2CO_3 (13.1 g, 40 mmol) and 10 mL anhydrous DMF in a period of 5 minutes. The suspension was stirred at 60 °C for 30 minutes, and 2,5-dibromo-1,3-difluorobenzene (2.7 g, 10 mmol) was injected dropwise into it within 5 minutes. The solution was stirred under reflux overnight. After completion (determined by TLC monitor), the reaction mixture was cooled down to room temperature, then poured into ice water. The mixture was filtered, and the solid was washed with water. The brown crude product was recrystallized with ethanol/ Hex to afford compound **3** as a white solid (5.6 g, 71%).

^{1}H NMR ($CDCl_3$, 400 MHz): The spectroscopic data are in accordance to reported literature;[3] δ 8.16 (d, J = 1.9 Hz, 4H), 7.75 (s, 2H), 7.53 (dd, J = 8.6, 1.9 Hz, 4H), 7.14

(d, J = 8.5 Hz, 4H), 1.48 (s, 36H).

BCzBN-Br

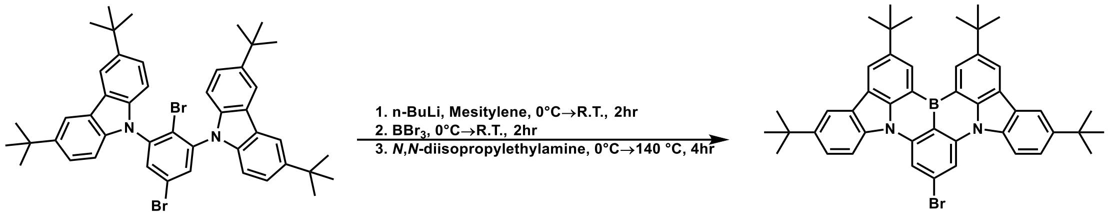

3   **BCzBN-Br**

Into an oven-dried 100 mL two-neck round bottom flask added compound **3** (2.7 g, 3.5 mmol) and anhydrous Xylene (30 mL) were added under N_2 atmosphere. The rection mixture was cooled to 0 °C for 10 minutes, *n*-BuLi in hexanes (2.63 mL, 1.6 M, 4.2 mmol) was added dropwise, then stirring at room temperature for 2 hours. BBr_3 (0.46 mL, 4.9 mmol) was slowly added to the solution at 0 °C. After returning to room temperature, the mixture was stirred for 2 hours. The suspension was cooled to 0 °C for 10 minutes, and DIPEA (1.22 mL, 7.0 mmol) was added dropwise. After stirring for 10 minutes, the reaction was warmed to 140 °C for 4 hours. Then the mixture was cooled to room temperature, quenched with water, and extracted with DCM. The organic layer was dried over Na_2SO_4 and filtered, and the solvent was removed under reduced pressure. The crude product **BCzBN-Br** was directly used without further purification.

BCzBN-CN

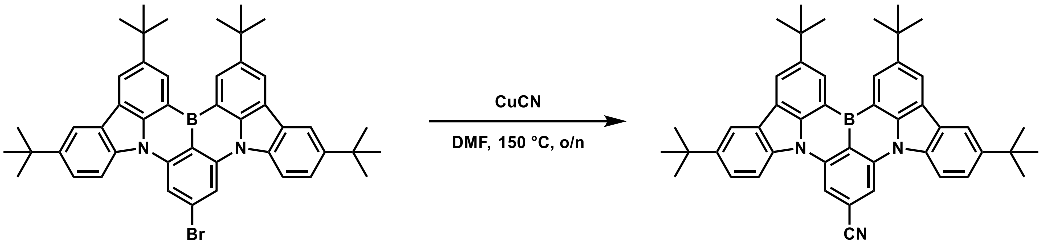

BCzBN-Br   **BCzBN-CN**

Into an oven-dried 25 mL two-neck round bottom flask was added **BCzBN-Br** (215.8 mg, 0.3 mmol), copper cyanide (CuCN, 40.3 mg, 0.45 mmol) and anhydrous DMF (10 mL) under N_2 atmosphere. The rection mixture was warmed to 150 ˚C and stirred for overnight. Then the mixture was cooled to room temperature, quenched with water, and extracted with ethyl acetate (EA). The organic layer was dried over Na_2SO_4 and filtered, and the solvent was removed under reduced pressure. The yellow crude product was purified by column chromatography (silica gel, 20% EA/Hex) to afford **BCzBN-CN** as a light-yellow solid (91.3 mg, 46 %).

^{1}H NMR (CDCl$_3$, 400 MHz): The spectroscopic data are in accordance to reported literature;[3] δ 9.07 (d, *J* = 1.9 Hz, 2H), 8.47 (d, *J* = 1.8 Hz, 2H), 8.41 (s, 2H), 8.29 – 8.22 (m, 4H), 7.68 (dd, *J* = 8.8, 2.1 Hz, 2H), 1.67 (s, 18H), 1.54 (s, 18H).

BCzBN-AC

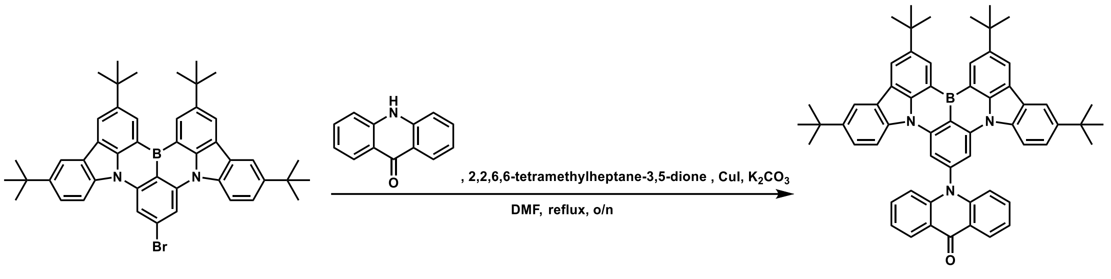

BCzBN-Br

BCzBN-AC

BCzBN-Br (1.1 g, 2.0 mmol), acridin-9(10*H*)-one (350.2 mg, 1.8 mmol), copper iodide (34.2 mg, 0.18 mmol), potassium carbonate (K_2CO_3, 272.7 mg, 2.0 mmol) and 2,2,6,6-tetramethyl-3,5-heptanedione (66.1 mg, 0.36 mmol) were suspended in anhydrous DMF (10 mL) into an oven-dried 50 mL two-neck round bottom flask under N_2 atmosphere. The reaction mixture was refluxed and stirred overnight. Then the mixture was cooled to room temperature, quenched with water, and extracted with EA. The organic layer was dried over Na_2SO_4 and filtered, and the solvent was removed under reduced pressure. The yellow crude product was purified by column chromatography (silica gel, 20% EA/Hex) to afford **BCzBN-AC** as a light-yellow solid (453.1 mg, 37 %).

^{1}H NMR (CDCl$_3$, 400 MHz): δ 9.21 (d, *J* = 1.9 Hz, 2H), 8.76 – 8.68 (m, 2H), 8.53 (d, *J* = 1.8 Hz, 2H), 8.32 (s, 2H), 8.26 (d, *J* = 2.1 Hz, 2H), 8.11 (d, *J* = 8.9 Hz, 2H), 7.57 – 7.49 (m, 4H), 7.35 (ddd, *J* = 8.0, 6.9, 1.0 Hz, 2H), 7.15 – 7.09 (m, 2H), 1.71 (s, 18H), 1.46 (s, 18H).

^{13}C NMR (CDCl$_3$, 101 MHz): δ 146.23, 146.20, 145.53, 143.07, 141.60, 137.96, 133.75, 129.97, 127.44, 127.20, 124.78, 123.99, 121.92, 121.87, 121.60, 121.28, 117.43, 117.01, 114.08, 108.68, 77.32, 77.00, 76.68, 35.27, 34.78, 32.16, 31.68.

HRMS (ESI-TOF): m/z for $C_{59}H_{57}BN_3O^+$ $[M+H]^+$, calc'd: 834.4599, found: 834.4592.

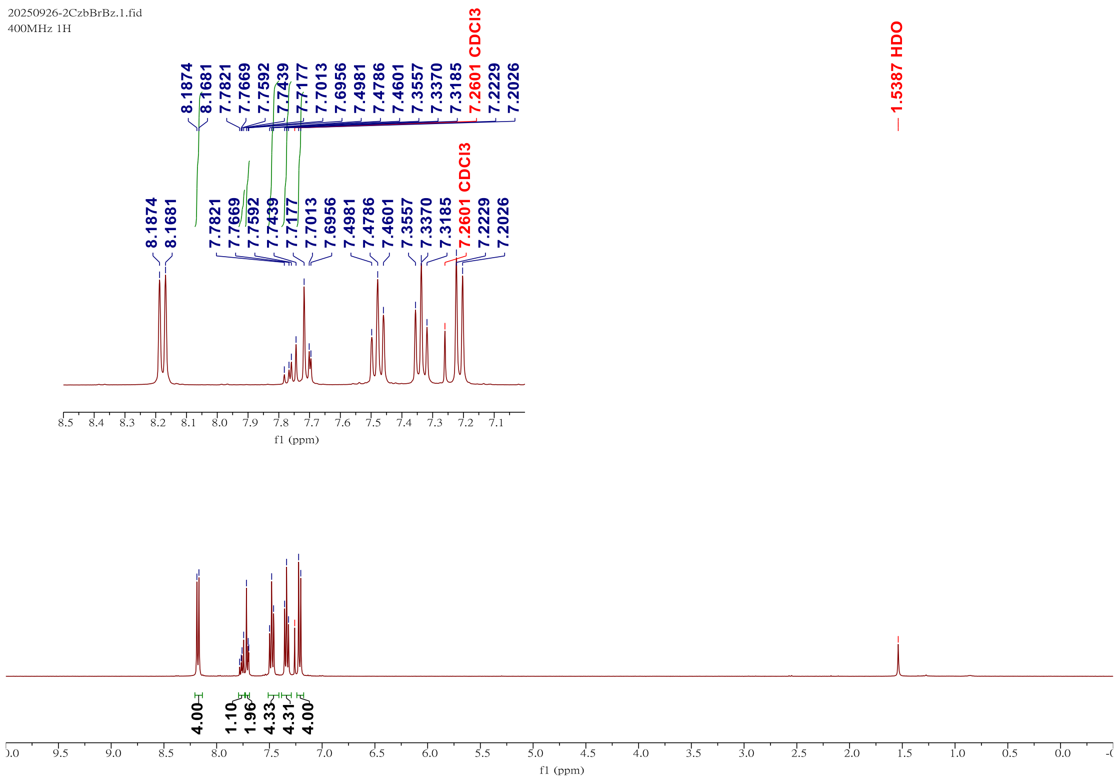

Figure S1. ¹H NMR spectrum of compound 1 ($CDCl_3$).

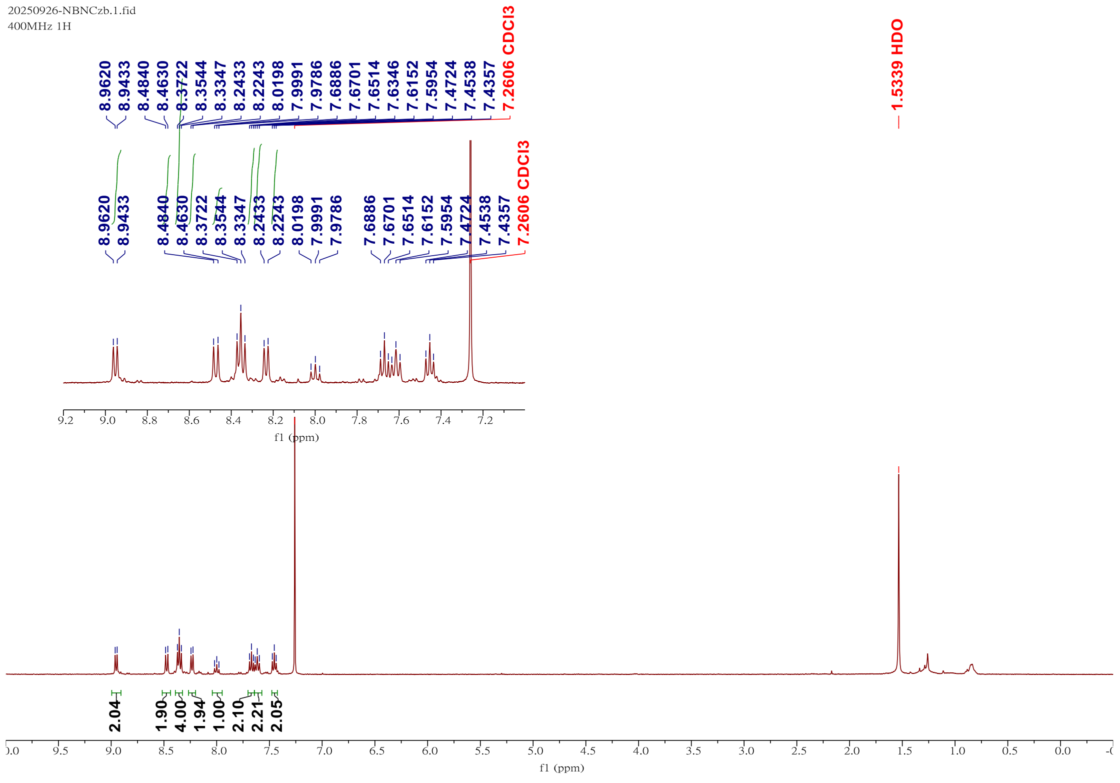

Figure S2. ¹H NMR spectrum of CzBN ($CDCl_3$).

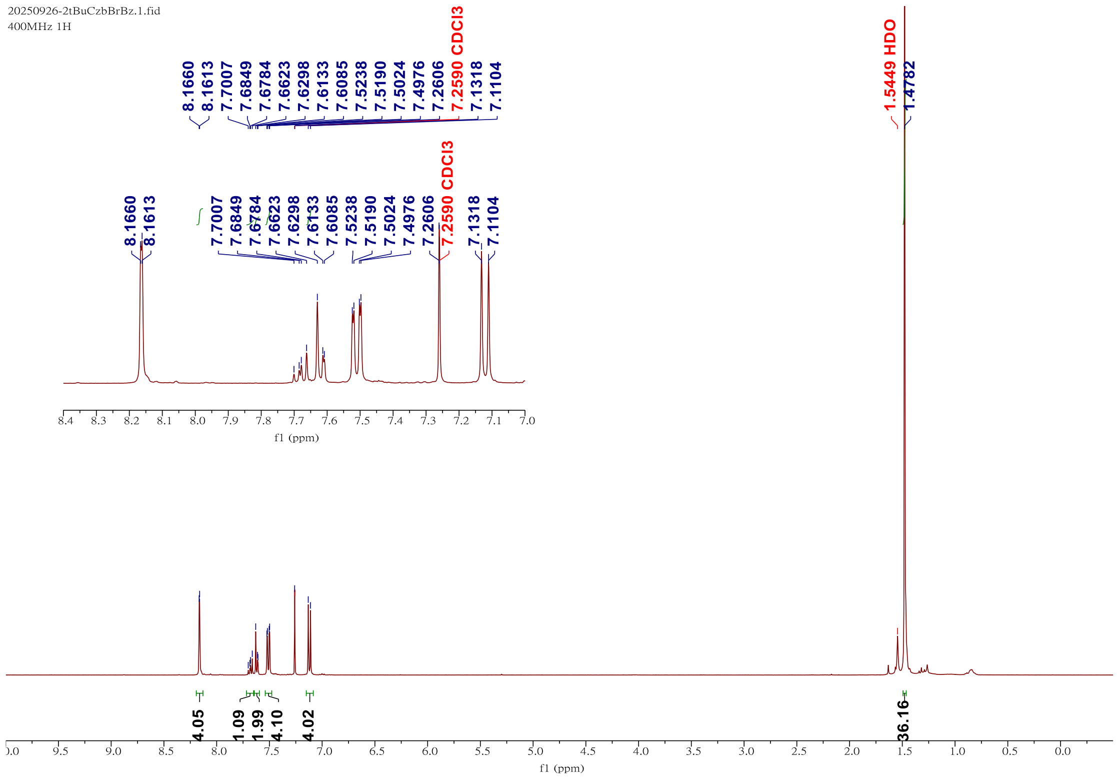

Figure S3. ^{1}H NMR spectrum of compound 2 ($CDCl_3$).

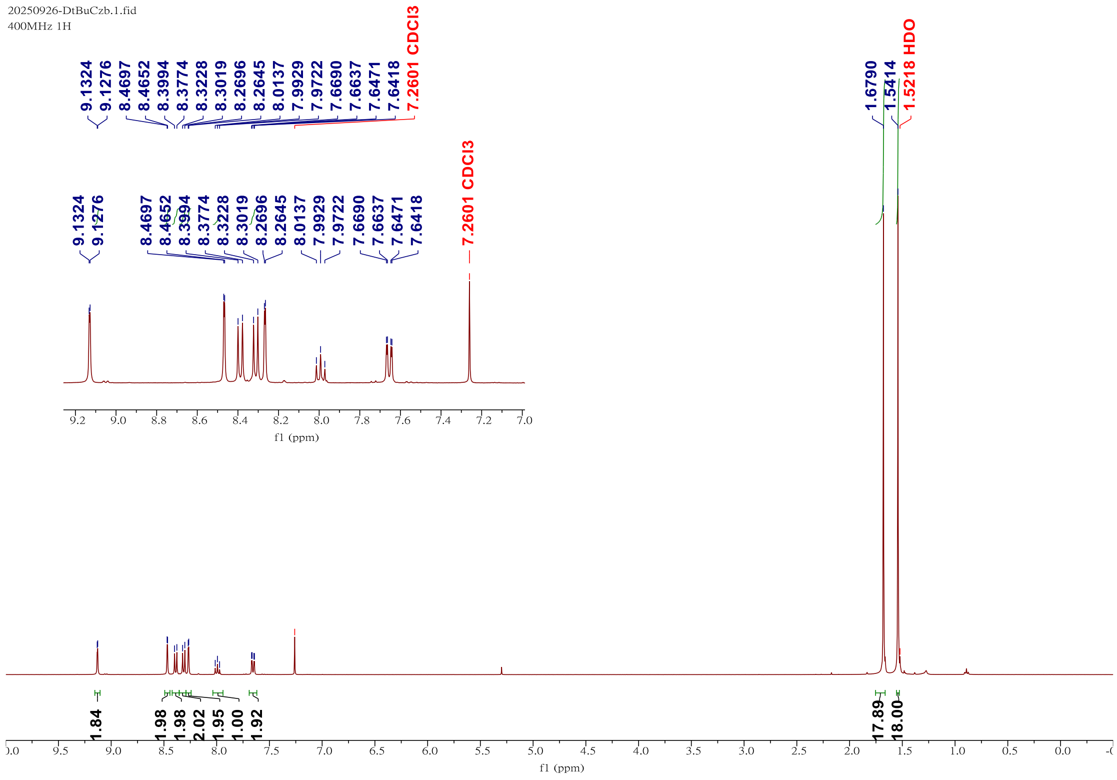

Figure S4. ^{1}H NMR spectrum of BCzBN ($CDCl_3$).

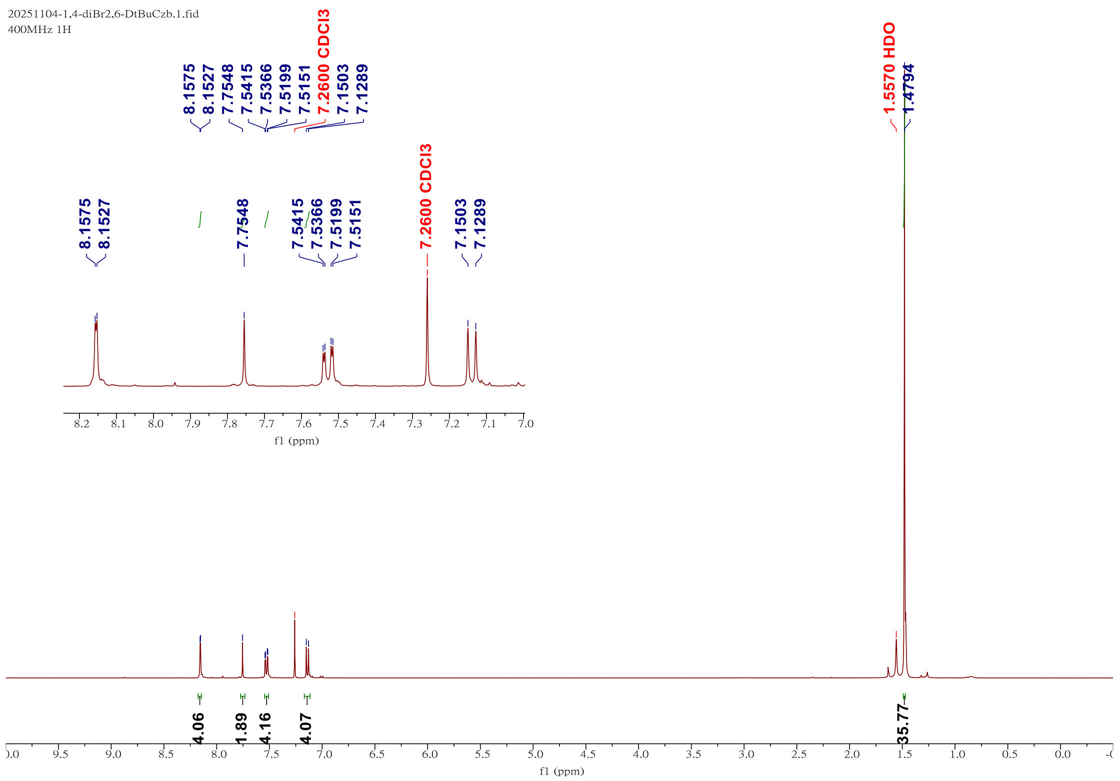

Figure S5. ^{1}**H NMR spectrum of compound 3 ($CDCl_3$).**

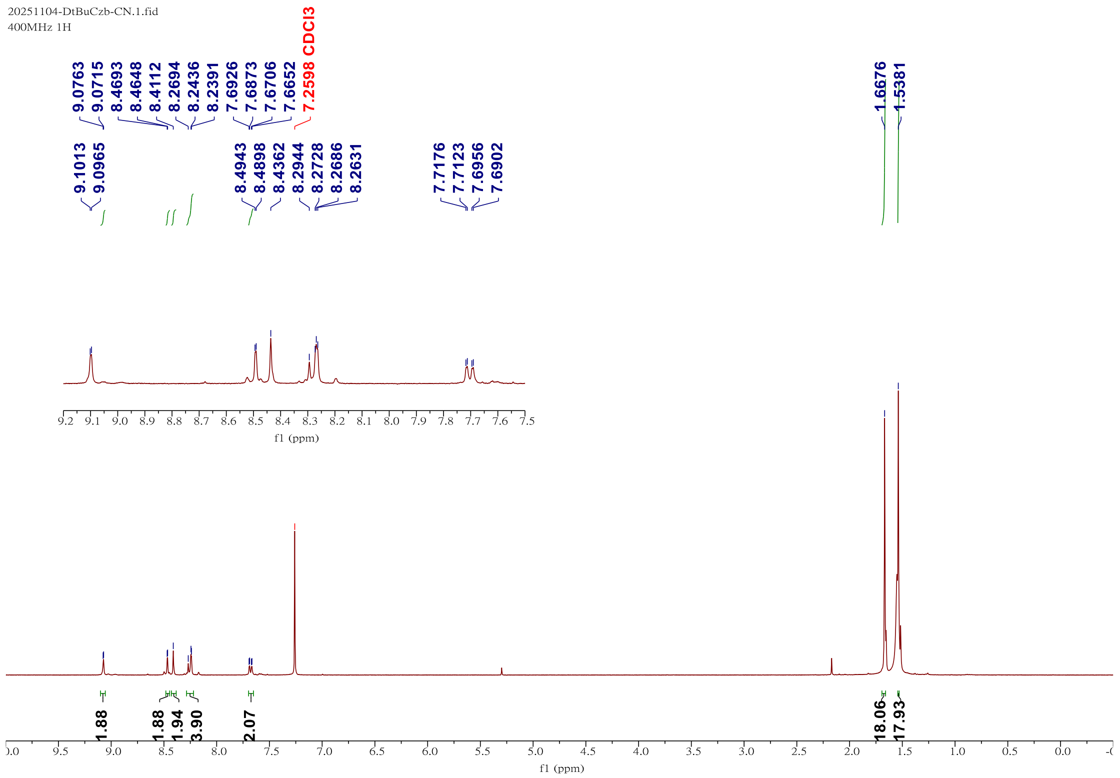

Figure S6. ^{1}**H NMR spectrum of BCzBN-CN ($CDCl_3$).**

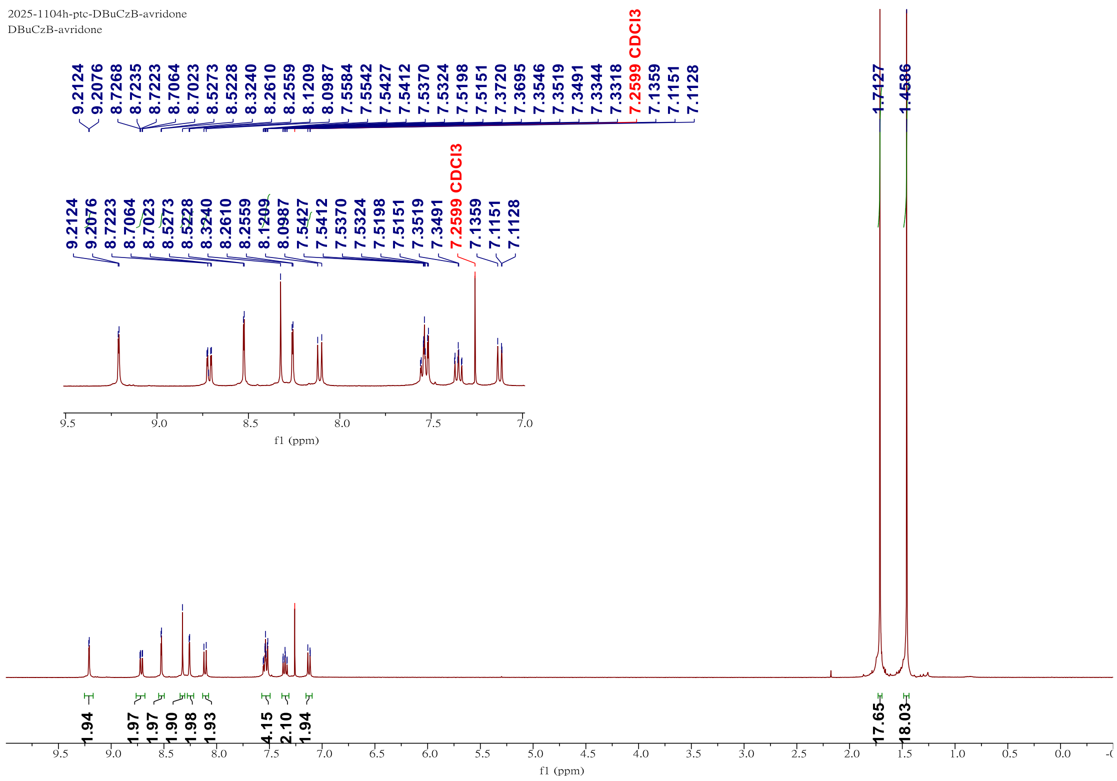

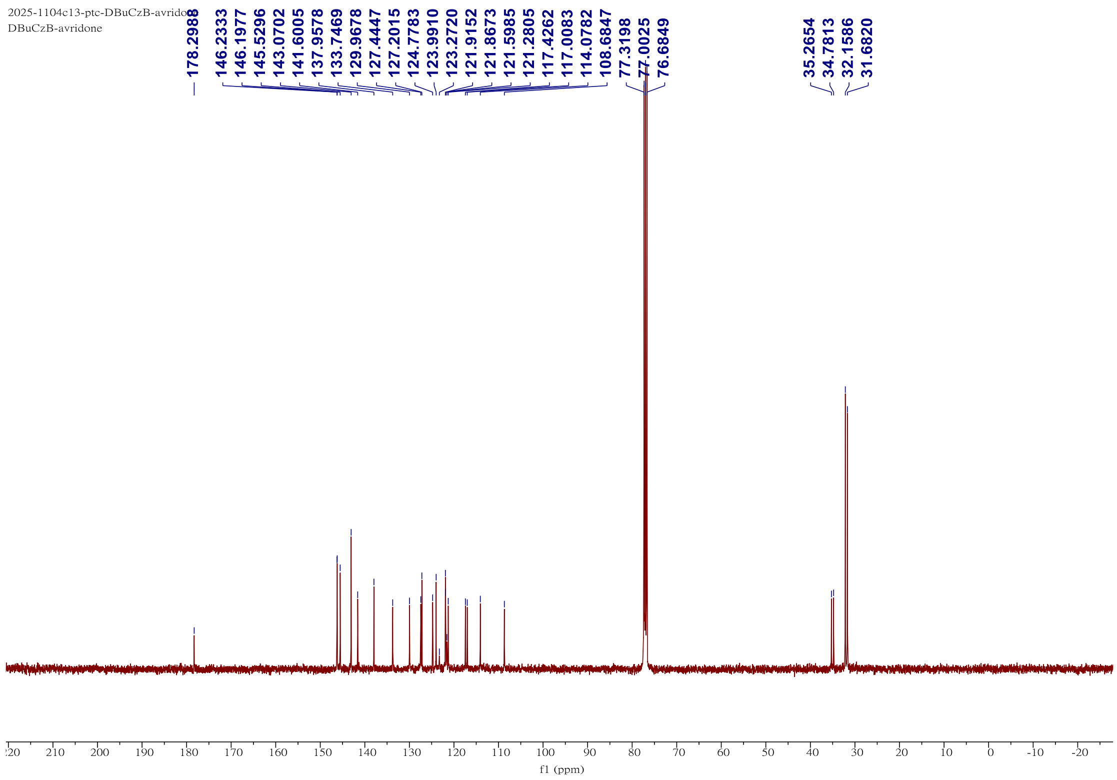

Figure S7. ^{1}H NMR and ^{13}C spectrum of BCzBN-AC ($CDCl_3$).

3. Photophysical Properties

Steady-state Spectra

Absorption spectra were recorded on a double-beam spectrophotometer (Hitachi UH5700). Steady-state excitation and emission spectra were recorded on a fluorescence spectrometer (Edinburgh FS980 and FS920). The dilute solutions of the samples (*ca.* 10^{-5}–10^{-6} M, unless otherwise noted) were measured in quartz cuvettes with 1 cm optical path length. Photoluminescence quantum yield (PLQY) of **CzBN** was determined via a relative method, using Coumarin 480 in methanol (Q.Y. = 0.87) as the standard. For the **3H$_2$O–CzBN** complex, the PLQY was determined relatively by using the initial **CzBN** solution (prior to water titration) as the reference and the final solution (with 140 μL water, see Figure 2 a,b) as the complex solution. All kinetic measurements were performed under degassed environment, achieved through three freeze-pump-thaw cycles (each cycle involved pumping for over 10 minutes, and the degassing efficiency was confirmed by a pressure gauge). Spectroscopic-grade solvents were purchased from J.T. Baker (dichloromethane), Thermo Fisher Scientific (toluene), and Merck (cyclohexane, ethyl acetate, tetrahydrofuran, and acetonitrile). THF was dried over calcium hydride overnight, followed by distillation. Secondary-deionized water was used throughout this study.

Lifetime Measurements

Time-resolved photoluminescence measurements were performed using time-correlated single photon counting (TCSPC) with time-resolution of 1.3 ns and multichannel scaling (MCS) techniques (Edinburgh FLS980) with time-resolution of 1.1 μs. The TCSPC measurements employed a picosecond pulsed diode laser (EPL-375, λ_{ex} = 377.8 nm; and EPL-295, λ_{ex} = 301.1 nm; Edinburgh Instruments) as the excitation light sources. The MCS measurements employed the third harmonic (TH, λ_{ex} = 355 nm) output of a nanosecond pulsed Nd:YAG laser (Surelite SLI-10, Continuum) as the excitation light source. The lifetimes from TCSPC measurements were obtained via reconvolution fitting using the built-in Fluoracle software (Edinburgh Instruments). For the MCS measurements, the data were fitted using a bi-exponential function (shown below) via the least-squares method in MATLAB software.

$$Intensity\ (t) = A_1 e^{\left(-\frac{t-t_0}{\tau_1}\right)} + A_2 e^{\left(-\frac{t-t_0}{\tau_2}\right)}$$

Here, A_1 and A_2 are pre-exponential factors, τ_1 and τ_2 are lifetimes, and t_0 is the time-zero determined by the rise of the IRF function (fixed during simulation).

Time-resolved Photoluminescence Spectra

Time-resolved Photoluminescence spectra were performed using tunable pulse laser (LS-2145-OPO, LOTIS TII) as excitation source (λ_{ex} = 450 nm, OPO mode; λ_{ex} = 355 nm, Nd:YAG TH mode) and the emission spectra were collected at perpendicular direction of laser pulse. The emission spectra of samples were resolved by grating monochromator (SP-2300i, Princeton Instruments) and recorded by an intensified charge-coupled device (ICCD, PI-MAX, Princeton Instruments).

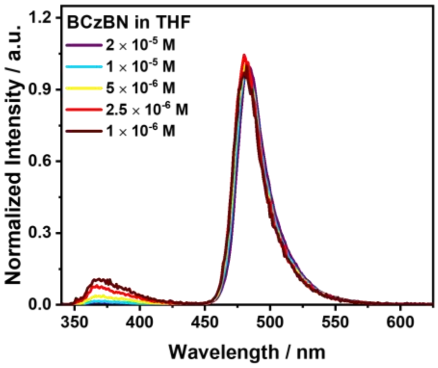

Figure S8. Normalized emission spectra of **BCzBN** in tetrahydrofuran at various concentrations. The excitation wavelength was 320 nm.

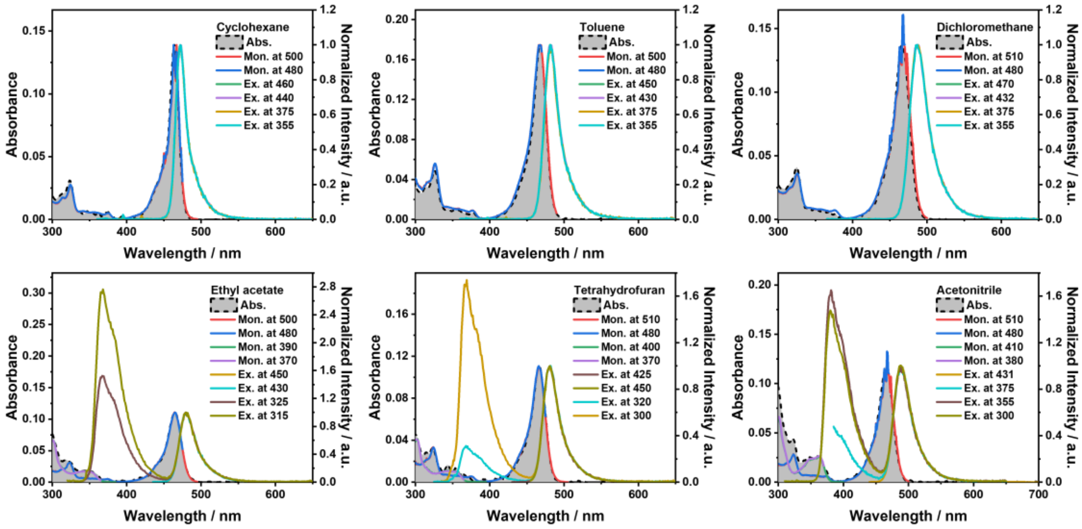

Figure S9. Steady-state spectra of **BCzBN** in various solvents.

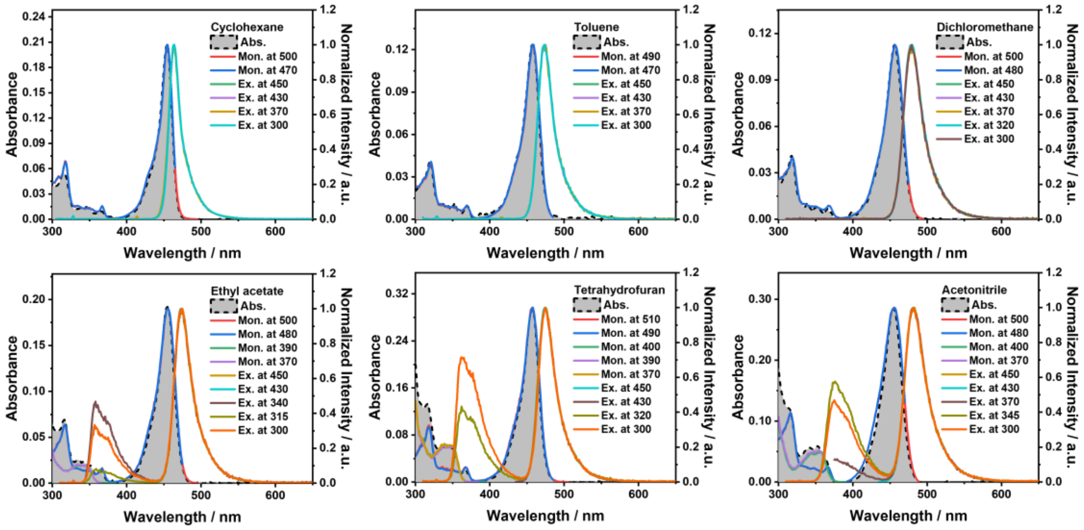

Figure S10. Steady-state spectra of **CzBN** in various solvents.

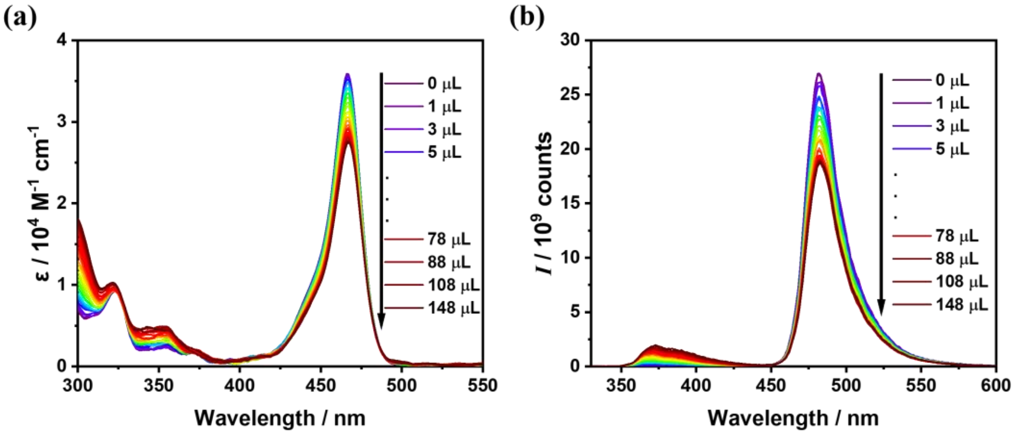

Figure S11. The spectral changes of **BCzBN** in (a) absorption and (b) fluorescence spectra (λ_{ex} = 320 nm) upon gradual addition of water (0–140 μL) into THF solution (10^{-5} M, 2.6 mL). Both absorbance and photoluminescence intensity were calibrated to account for volume variations during titration, expressed as extinction coefficient (ε) and molar PL intensity (I). Note: The counts in y-axis have been calibrated by the dilution of sample concentration due to the added water volume.

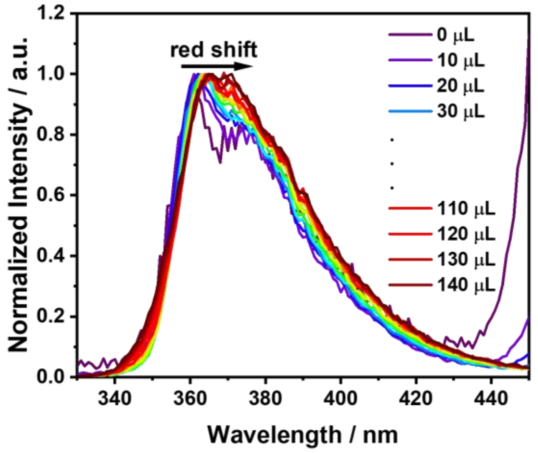

Figure S12. Normalized PL spectra of **CzBN** upon gradual addition of water (0–140 μL) to a THF solution (10^{-5} M, 2.6 mL). The excitation wavelength was 320 nm.

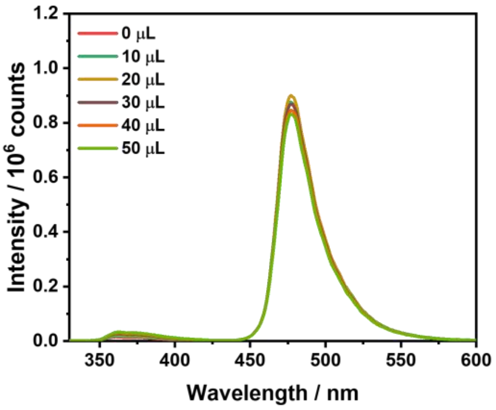

Figure S13. Emission spectra of **CzBN** (10^{-5} M, 2.6 mL, THF) with butylated hydroxytoluene (BHT, *ca.* 200 ppm) upon gradual addition of water (0–50 μL). The excitation wavelength was 320 nm. The spectral change is significantly suppressed in presence of BHT compared to Figure 2.

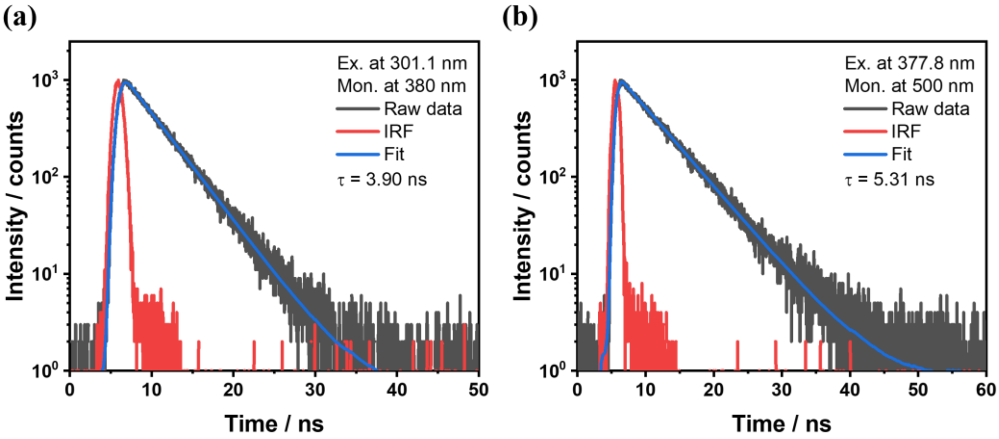

Figure S14. Time-resolved fluorescence profiles of **CzBN** (10^{-5} M, 2.6 mL, THF) with 2 M of water, monitored at (a) 380 nm and (b) 500 nm, corresponding to the emissions of **3H$_2$O–CzBN** and **CzBN**, respectively. The data were collected using the TCSPC technique and analyzed by reconvolution fitting.

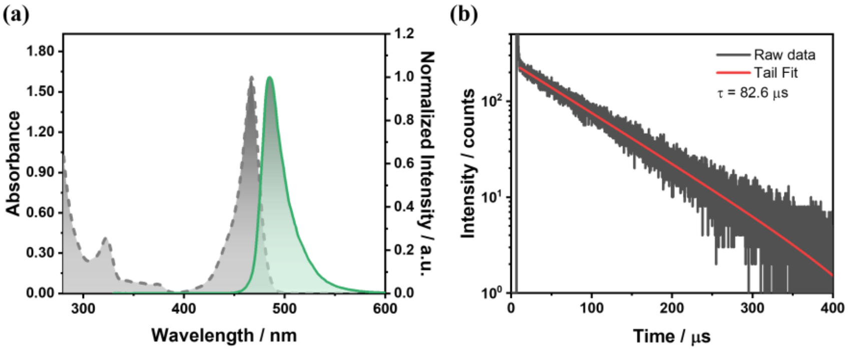

Figure S15. (a) Absorption (grey dashed line, shaded) and emission (green solid line, shaded; λ_{ex} = 320 nm) spectra of **CzBN** in anhydrous THF. (b) Time-resolved fluorescence profile of the same solution recorded using the MCS method. Data was collected at 500 nm.

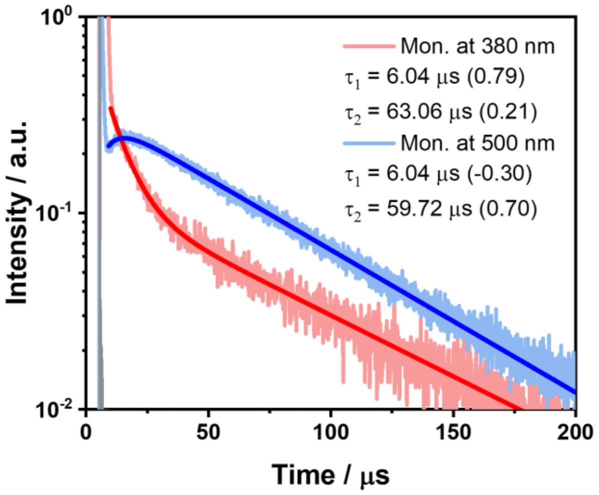

Figure S16. Time-resolved fluorescence profiles of **CzBN** (10^{-5} M, 2.6 mL, THF) with 0.6 M of water, monitored at 380 nm (red) and 500 nm (blue). Both kinetics measurements were recorded using the MCS method and simultaneously fitted with a bi-exponential function, where the τ_1 values for both profiles were constrained to be identical.

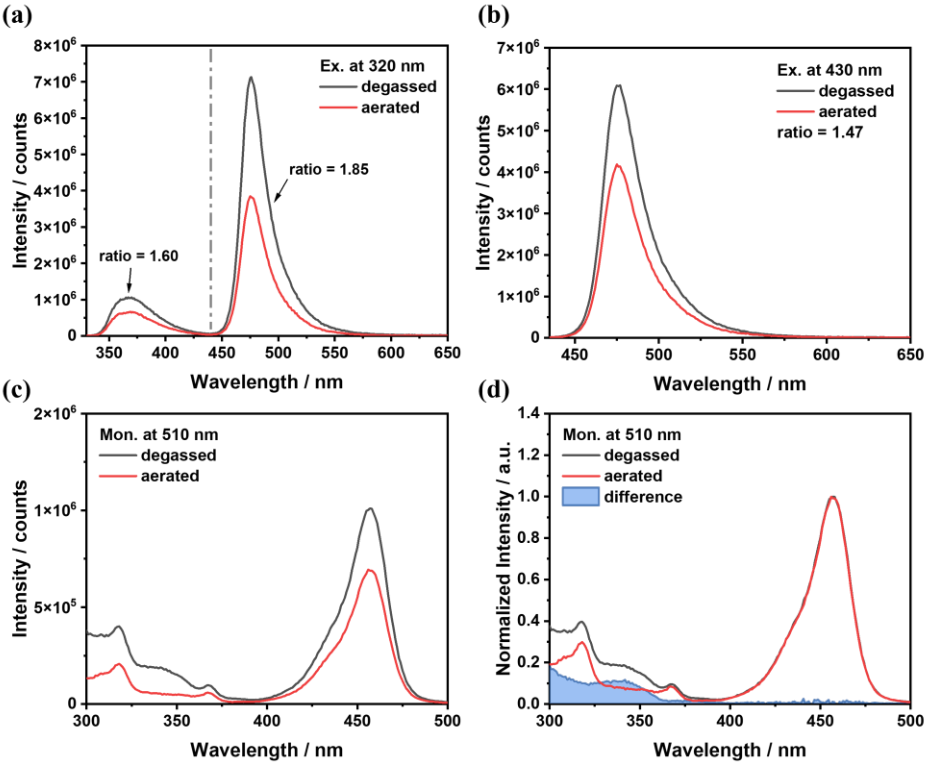

Figure S17. Photoluminescence spectra of **CzBN** (10^{-5} M, 2.6 mL, THF) containing 0.6 M water under (a) λ_{ex} = 320 nm and (b) λ_{ex} = 430 nm excitation, as well as (c) excitation and (d) normalized excitation spectra monitored at 510 nm. Black and red lines correspond to degassed and aerated conditions, respectively. The ratio values in

(a) and (b) represent the integrated PL intensity ratio under different conditions, where the integration ranges of 330–436 nm and 437–650 nm, corresponding to each emission band, are indicated by dashed line in (a). The difference spectra, highlighted in blue in (d), correspond well to the assigned excitation band of **3H$_2$O–CzBN**.

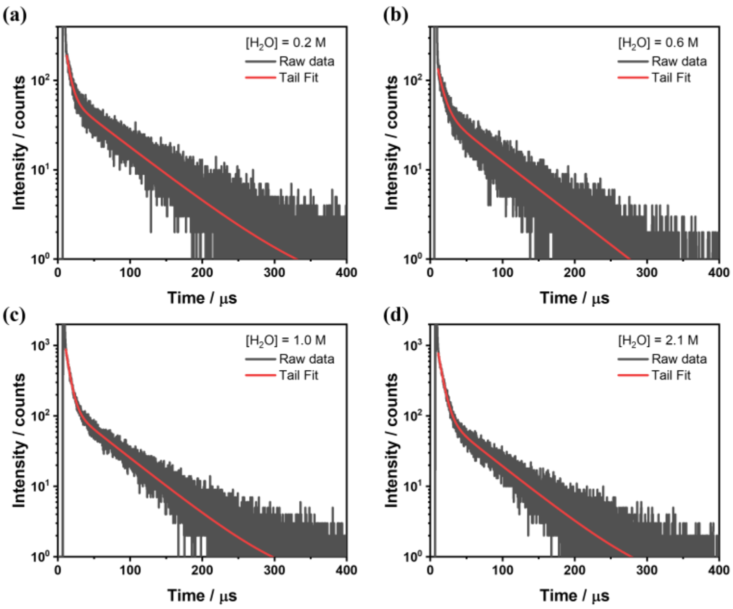

Figure S18. Time-resolved fluorescence profiles of **CzBN** (10^{-5} M, 2.6 mL, THF) with various water concentrations: (a) 0.2 M, (b) 0.6 M, (c) 1.0 M, and (d) 2.1 M, monitored at 380 nm. All kinetic measurements were recorded using the MCS method and fitted with a bi-exponential function. The fitting parameters are summarized in Table S3.

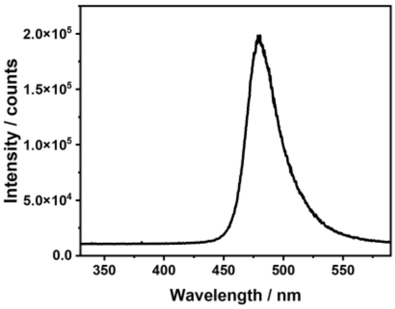

Figure S19. Delayed emission spectrum of **CzBN** (10^{-5} M, 2.6 mL, THF) with 1.1 M water under 450 nm excitation (selectively exciting **CzBN**). The spectrum was recorded using ICCD with a gate delay of 5 μs and a gate width of 400 μs. The result indicates that the excited-state association pathway (**CzBN** + 3H$_2$O → **3H$_2$O–CzBN**) is unavailable.

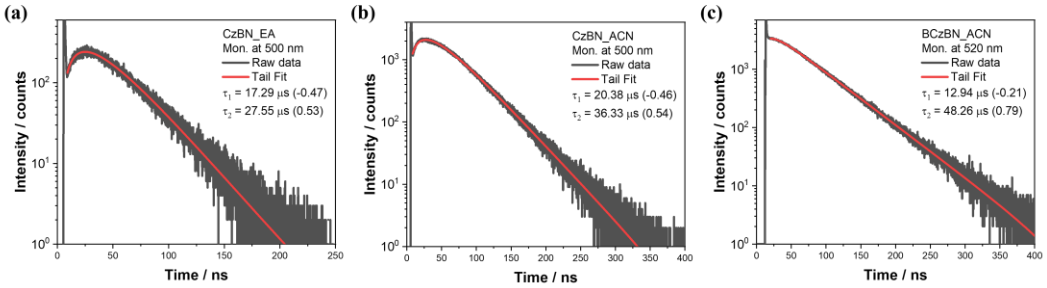

Figure S20. Time-resolved fluorescence profiles of (a) **CzBN** in unprocessed EA, (b) **CzBN** in unprocessed ACN, and (c) **BCzBN** in unprocessed ACN. The data were collected using the MCS technique and fitted with a bi-exponential function.

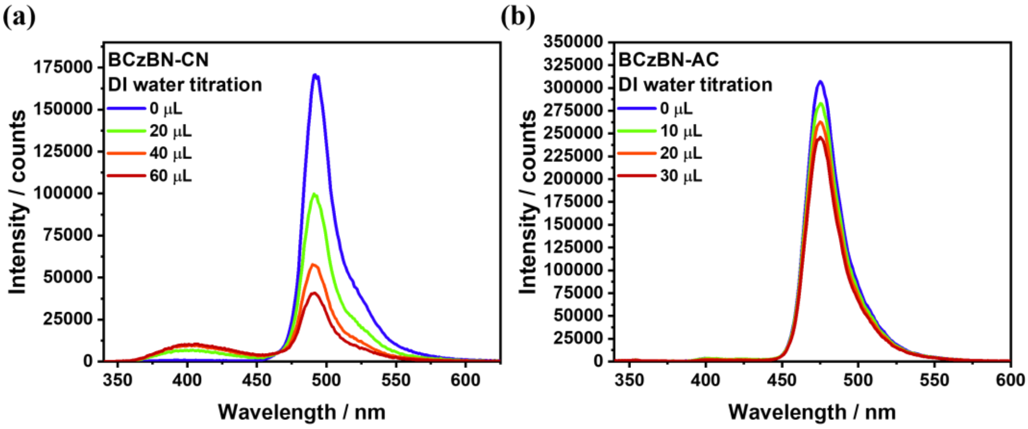

Figure S21. The spectral changes of (a) **BCzBN-CN** and (b) **BCzBN-AC** fluorescence spectra (λ_{ex} = 320 nm) upon gradual addition of water into THF solution (10^{-5} M, 2.6 mL).

Table S1. Organic solvent properties and observation of dual emission

Solvent	Dielectric Constant (20°C)[a]	Solubility of water in solvent (25°C, %w/w)[a]	Emission band at 380 nm[b]
Cyclohexane	2.01	0.01	X
Toluene	2.38	0.033	X
Ethyl acetate	6.02	3.3	O
Tetrahydrofuran	7.6	miscible	O
Dichloromethane	9.1	0.2	X
Acetonitrile	37.5	miscible	O

Note: [a]All the constants are extracted from Handbook of organic solvent properties, Ref ([4]). [b]If **BCzBN** and **CzBN** shows 380 nm emission band when exciting wavelength is < 370 nm, record as "O"; otherwise, record as "X". For more detailed data, see Figure S9 and S10.

Table S2. Kinetic fitting results of **CzBN** at various detection wavelengths

λ_{mon}	τ_2 / μs (prefactor)	τ_3 / μs (prefactor)
380	7.22 (0.80)	67.93 (0.20)
500	4.18 (-0.41)	60.58 (0.59)

Notice: The sample was prepared in THF (10^{-5} M, 2.6 mL) containing 0.6 M water. All kinetic measurements were recorded using the MCS method and fitted with a bi-exponential function.

Table S3. Kinetic fitting results of **CzBN** at various water concentrations

[H_2O]	τ_2 / μs (prefactor)	τ_3 / μs (prefactor)
0.2 M	5.99 (0.67)	69.77 (0.33)
0.6 M	7.22 (0.80)	67.93 (0.20)
1.0 M	5.59 (0.85)	53.54 (0.15)
2.1 M	6.48 (0.87)	53.55 (0.13)

Notice: The sample was prepared in THF (10^{-5} M, 2.6 mL). All kinetic measurements were recorded at 380 nm using the MCS method and fitted with a bi-exponential function.

Fitting of binding constant (*K*):

Given a reaction model between **CzBN** and *m* equivalents water:

$$CzBN + mH_2O \underset{}{\overset{K}{\rightleftharpoons}} mH_2O\text{-}CzBN$$

$$K = \frac{[m\mathrm{H_2O\text{-}CzBN}]}{[\mathrm{CzBN}][\mathrm{H_2O}]^m} \qquad \text{Equation S1}$$

$$[\mathrm{CzBN}] = \frac{n^0_{\mathrm{CzBN}} - \delta}{V_t} \qquad \text{Equation S2}$$

$$[m\mathrm{H_2O\text{-}CzBN}] = \frac{\delta}{V_t} \qquad \text{Equation S3}$$

$$[\mathrm{H_2O}] = \frac{n^0_{\mathrm{H2O}} - m\delta}{V_t} \qquad \text{Equation S4}$$

Where is $[m\mathrm{H_2O\text{-}CzBN}]$ concentration of complex, $[\mathrm{CzBN}]$ is concentration of **CzBN**, $[\mathrm{H_2O}]$ is concentration of water, in dynamic equilibrium. *K* is the binding constant, V_t is total volume of solution, n^0_{CzBN} and n^0_{H2O} are initial moles of **CzBN** and water, respectively. The reaction equilibrium can be expressed as the following equivalent equilibrium:

$$[\mathrm{CzBN}] = \frac{n^0_{\mathrm{CzBN}} - n^0_{\mathrm{CzBN}} + \delta}{V_t} = \frac{\delta}{V_t} \qquad \text{Equation S5}$$

$$[m\mathrm{H_2O\text{-}CzBN}] = \frac{n^0_{\mathrm{CzBN}} - \delta}{V_t} \qquad \text{Equation S6}$$

$$[\mathrm{H_2O}] = \frac{n^0_{\mathrm{H2O}} - n^0_{\mathrm{CzBN}} + m\delta}{V_t} \approx \frac{n^0_{\mathrm{H2O}}}{V_t} \qquad \text{Equation S7}$$

Where the n^0_{H2O} is much larger than n^0_{CzBN} and $m\delta$, so equation S7 can be simplified as $[\mathrm{H_2O}] \approx (n^0_{\mathrm{H2O}})/V_t$. By substituting Equation. S7 into Equation S1, we obtain:

$$K = \frac{\left(\frac{n^0_{\mathrm{CzBN}} - \delta}{V_t}\right)}{\left(\frac{\delta}{V_t}\right)\left(\frac{n^0_{\mathrm{H2O}}}{V_t}\right)^m} = \frac{(n^0_{\mathrm{CzBN}} - \delta){V_t}^m}{\delta(n^0_{\mathrm{H2O}})^m} \qquad \text{Equation S8}$$

After rearrangement, the following expression is obtained:

$$\delta = \frac{n^0_{\mathrm{CzBN}}{V_t}^m}{n^0_{\mathrm{H2O}}K + {V_t}^m} \qquad \text{Equation S9}$$

$$[\mathrm{CzBN}] = \frac{\delta}{V_t} = \frac{n^0_{\mathrm{CzBN}}{V_t}^{m-1}}{n^0_{\mathrm{H2O}}K + {V_t}^m} \qquad \text{Equation S10}$$

According to the Beer-Lambert Law, the absorbance (Abs) is given by:

$$Abs_{CzBN} = \varepsilon b[\mathrm{CzBN}] = \varepsilon b \frac{n^0_{\mathrm{CzBN}} {V_t}^{m-1}}{n^0_{\mathrm{H2O}} K + {V_t}^m} \qquad \text{Equation S11}$$

Here, the ε is molar extinction coefficient and b is optical path length (1 cm), both of which are constant. Initial amount of **CzBN**, n^0_{CzBN}, is also a constant and equal to 10^{-5} M × 2.6×10^{-3} L. The remaining parameters can be reduced to the only variable in the titration experiment, namely the volume of water added to the solution (V_{H2O}).

$$V_t = 2.6 \times 10^{-3} + V_{H2O} \qquad \text{Equation S12}$$

$$n^0_{\mathrm{H2O}} = \frac{V_{H2O} \times 10^3}{18} \qquad \text{Equation S13}$$

Similarly, the stoichiometric ratio can also be derived from fitting the titration plots of photoluminescence (PL) intensity, as shown below.

$$\frac{[m\mathrm{H_2O\text{-}CzBN}]}{[\mathrm{CzBN}]} = \frac{F_{complex}/(\varepsilon_{complex}\phi_{complex})}{F_{CzBN}/(\varepsilon_{CzBN}\phi_{CzBN})} \qquad \text{Equation S14}$$

Where $F_{complex}$ and F_{CzBN} represent PL intensities of the complex and **CzBN**, respectively; $\varepsilon_{complex}$ and ε_{CzBN} denote their molar extinction coefficients at the excitation wavelength; $\phi_{complex}$ and ϕ_{CzBN} are the corresponding quantum yields. Notice that both of ε and ϕ for each species remain constant during the titration experiment, as the excitation wavelength is fixed.

By substituting equation S14 into equation S1 and rearrange, we obtain:

$$K = \frac{F_{complex}/(\varepsilon_{complex}\phi_{complex})}{(F_{CzBN}/(\varepsilon_{CzBN}\phi_{CzBN}))[\mathrm{H_2O}]^m} \qquad \text{Equation S15}$$

$$\frac{F_{complex}}{F_{CzBN}} = \frac{(\varepsilon_{complex}\phi_{complex})K}{(\varepsilon_{CzBN}\phi_{CzBN})}[\mathrm{H_2O}]^m = C \times [\mathrm{H_2O}]^m \qquad \text{Equation S16}$$

Where $C = ((\varepsilon_{complex}\phi_{complex})K)/(\varepsilon_{CzBN}\phi_{CzBN})$ is a constant in the equation. Based on the above discussion, the titration plots of absorbance at 457 nm ($A_{457\mathrm{nm}}$, the absorbance peak of **CzBN**) were fitted using $\varepsilon_{457} = 1.25 \times 10^5$ $\mathrm{M^{-1}cm^{-1}}$, which was determined from a known absorbance 1.25 at a concentration of 10^{-5} M. The PL intensity ratio was also fitted using equation S16, where the PL intensities at 370 and 475 nm (denoted as $F_{370\mathrm{nm}}$ and $F_{475\mathrm{nm}}$) were selected to represent the complex and

CzBN, respectively. Notice that only the first six data points were used in the fitting to minimize deviation (see Figure 2c).

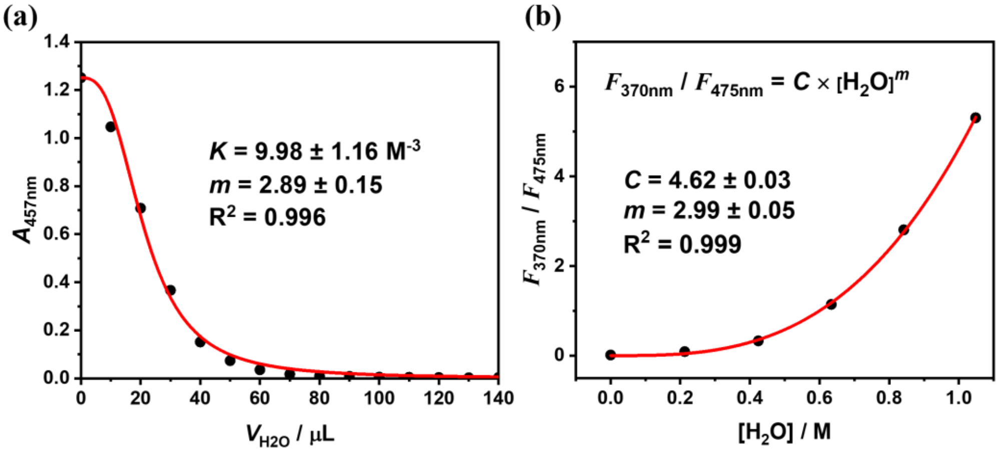

Figure S22. (a) Fitting result of the titration plots of absorbance at 457 nm (A_{457nm}) versus volume of added water (V_{H2O}) by using equation S11. (b) Fitting result of the titration plots of PL intensity ratio versus water concentration by using equation S16. Black dots represent the experimental data, while red lines represent the fitted curves. Both fitting results yield a stoichiometric coefficient of $m = 3$.

From the fitting result, we deduced a stoichiometric ratio of 1 (**CzBN**) to 3 (H_2O) molecules; therefore, we fixed $m = 3$ in the following fitting to obtain a more accurate binding constant, K.

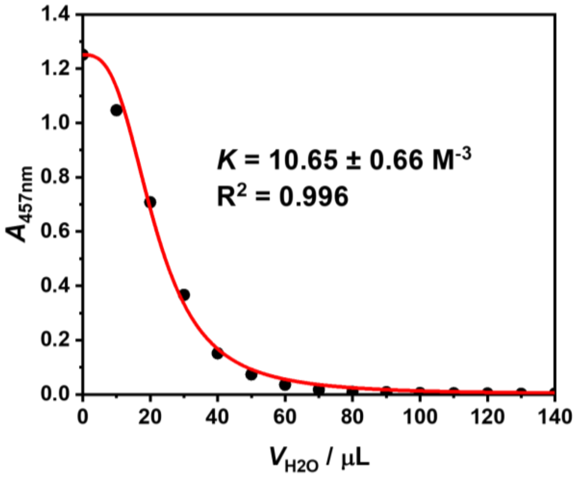

Figure S23. Fitting result of the titration plots of absorbance at 457 nm (A_{475nm}) versus volume of water (V_{H2O}) by using equation S11, using $m = 3$ as fixed parameter. The fitting result yields binding constant, $K = 10.65 \pm 0.66$ M^{-3}.

4. Computational Section

The calculations were performed with the Gaussian 16 package, using Density Function Theory (DFT) and Time-Dependent DFT (TD-DFT) methods with ωB97XD/6-31+G(d,p) scrf (solvent=tetrahydrofuran, polarizable continuum model (PCM)).[5-7]

Table S4. Computed optical excitations and molecular orbital contributions for **CzBN** and **BCzBN** with different numbers (*n*) of water molecules at S_0-optimized structure.

	n	no.	E/eV	λ/nm	f	Contribution	weight
CzBN / nH_2O	0	S_1	3.34	371.4	0.6830	HOMO→LUMO	91.8%
		S_2	4.05	306.3	0.0405	HOMO-1→LUMO	63.0%
						HOMO→LUMO+1	20.3%
		T_1	2.81	441.7	0.0000	HOMO→LUMO	66.1%
		T_2	3.03	408.7	0.0000	HOMO-2→LUMO+1	29.1%
						HOMO-1→LUMO	26.3%
						HOMO-3→LUMO	14.6%
	1	S_1	3.32	373.1	0.6604	HOMO→LUMO	91.8%
		S_2	4.04	307.1	0.0389	HOMO-1→LUMO	51.0%
						HOMO→LUMO+1	20.0%
						HOMO-2→LUMO	13.4%
		T_1	2.80	442.2	0.0000	HOMO→LUMO	68.6%
		T_2	3.03	409.8	0.0000	HOMO-1→LUMO	29.1%
						HOMO-1→LUMO+1	19.0%
	2	S_1	3.29	376.3	0.6496	HOMO→LUMO	92.1%
		S_2	4.04	307.3	0.0380	HOMO-1→LUMO	63.3%
						HOMO→LUMO+1	19.6%
		T_1	2.79	445.0	0.0000	HOMO→LUMO	73.1%
		T_2	3.01	411.7	0.0000	HOMO-2→LUMO	39.7%
						HOMO-1→LUMO+1	18.3%
						HOMO→LUMO	13.8%
	3 (bound)	S_1	4.17	297.2	0.4196	HOMO→LUMO	67.3%
						HOMO-1→LUMO+1	19.8%
		S_2	4.30	288.1	0.0354	HOMO-1→LUMO	42.0%
						HOMO→LUMO+1	43.1%
		T_1	3.17	391.4	0.0000	HOMO-2→LUMO	34.2%
						HOMO-3→LUMO+1	28.8%
		T_2	3.18	389.8	0.0000	HOMO-3→LUMO	31.5%
						HOMO-2→LUMO+1	30.7%
		T_3	3.40	364.4	0.0000	HOMO→LUMO+2	32.4%
		T_4	3.58	346.5	0.0000	HOMO→LUMO	47.7%
						HOMO-1→LUMO+1	24.1%
		S_1	3.34	371.4	0.6443	HOMO→LUMO	91.8%

		S_2	4.04	306.8	0.0399	HOMO-1→LUMO	60.9%
						HOMO→LUMO+1	19.8%
	3 (un-bound)	T_1	2.81	440.7	0.0000	HOMO→LUMO	66.9%
		T_2	3.02	410.4	0.0000	HOMO-1→LUMO	24.0%
						HOMO-2→LUMO+1	14.1%
						HOMO-1→LUMO+1	12.1%
		S_1	3.29	377.4	0.7579	HOMO→LUMO	91.5%
		S_2	3.97	312.2	0.0413	HOMO-1→LUMO	60.4%
						HOMO→LUMO+1	20.3%
	0	T_1	2.76	449.9	0.0000	HOMO→LUMO	71.0%
		T_2	2.97	416.9	0.0000	HOMO-1→LUMO	24.3%
						HOMO-1→LUMO+1	16.4%
						HOMO-2→LUMO	13.1%
		S_1	4.12	300.7	0.4494	HOMO→LUMO	66.5%
						HOMO-1→LUMO+1	20.3%
**BCzBN** / nH_2O		S_2	4.25	291.8	0.0360	HOMO-1→LUMO	44.1%
						HOMO→LUMO+1	41.0%
		T_1	3.15	393.9	0.0000	HOMO-2→LUMO	33.7%
	3 (bound)					HOMO-3→LUMO+1	24.4%
		T_2	3.16	392.3	0.0000	HOMO-3→LUMO	29.7%
						HOMO-2→LUMO+1	27.6%
		T_3	3.39	365.7	0.0000	HOMO→LUMO+2	30.8%
						HOMO-1→LUMO	10.7%
		T_4	3.52	352.4	0.0000	HOMO→LUMO	47.2%
						HOMO-1→LUMO+1	23.9%

Note on the Assignment of Calculated Triplet State

As summarized in Table S4, a systematic discrepancy exists between the experimental excitation energies and the values obtained via TD-DFT calculations. Consistent with literature reports on multi-resonance (MR) emitters, TD-DFT tends to significantly overestimate excitation energies.

For the unbound emitters (e.g., free **CzBN** and **BCzBN**), the T_1 state shares the same electronic character (HOMO→LUMO) as the S_1 state, with a calculated $\Delta E_{S\text{-}T}$ of approximately 0.5 eV. However, in the case of water-mediated complexes (**$3H_2O$–CzBN** and **$3H_2O$–BCzBN**), the corresponding triplet state with the same electronic character as S_1 is found to be T_4, with an energy gap of approximately 0.6 eV.

Due to the intrinsically higher excitation energy of these water-complexes, the calculation deviations in TD-DFT likely reorder the states, causing the "physically true" T_1 state to appear at a higher calculated index. Consequently, we have assigned the calculated T_4 state, which exhibits a $\Delta E_{S\text{-}T}$ (0.6 eV) and similar electronic properties to the S_1 state, as the physical relevant T_1 state that has undergone a significant blue-shift due to TD-DFT error.

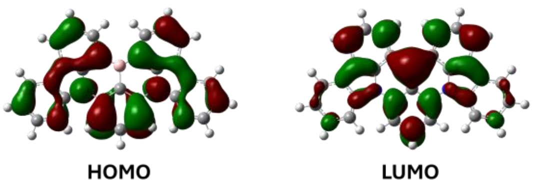

Figure S24. Frontier molecular orbitals associated with S_0-S_1 optical transitions of **CzBN** at S_0-optimized structure.

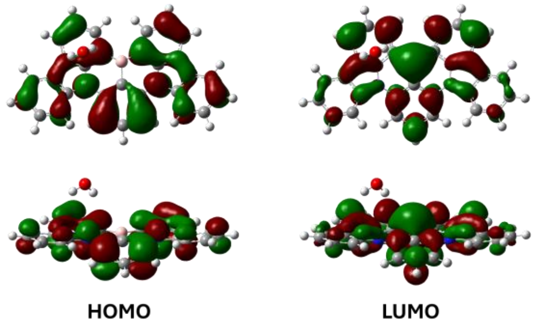

Figure S25. Frontier molecular orbitals associated with S_0-S_1 optical transitions of **CzBN / H_2O** at S_0-optimized structure. First row shows top view, and the other shows side view.

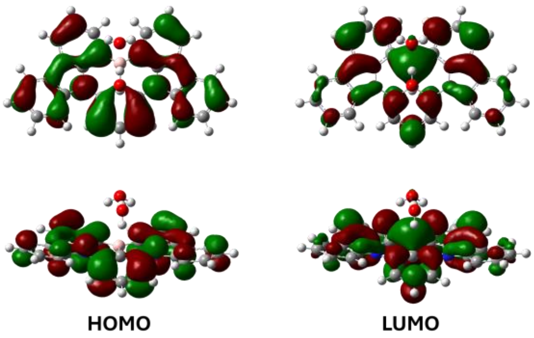

Figure S26. Frontier molecular orbitals associated with S_0-S_1 optical transitions of **CzBN / $2H_2O$** at S_0-optimized structure. First row shows top view, and the other shows side view.

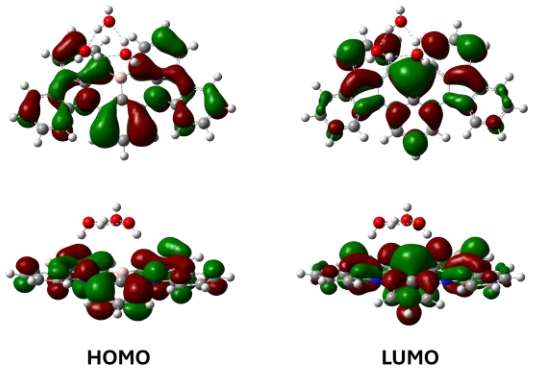

Figure S27. Frontier molecular orbitals associated with S_0-S_1 optical transitions of **CzBN / 3H$_2$O** (unbound) at S_0-optimized structure. First row shows top view, and the other shows side view.

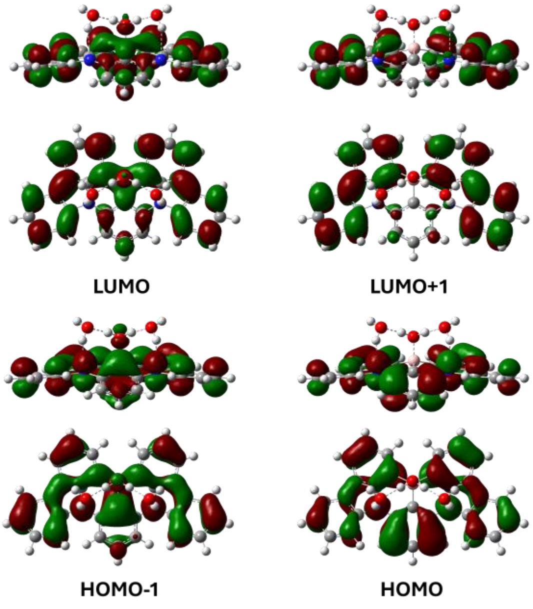

Figure S28. Frontier molecular orbitals associated with S_0-S_1 optical transitions of **3H$_2$O–CzBN** at S_0-optimized structure. First row shows top view, and the other shows side view.

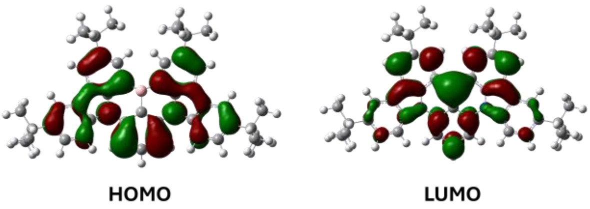

Figure S29. Frontier molecular orbitals associated with S_0-S_1 optical transitions of **BCzBN** at S_0-optimized structure.

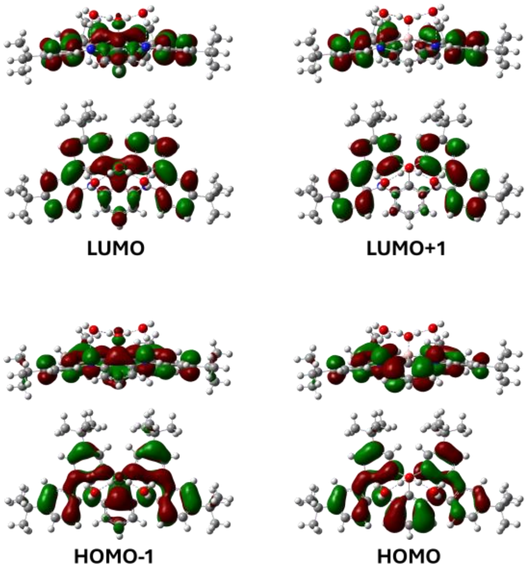

Figure S30. Frontier molecular orbitals associated with S_0-S_1 optical transitions of **3H$_2$O–BCzBN** at S_0-optimized structure. First row shows top view, and the other shows side view.

5. References

(1) Yan, X.; Li, Z.; Wang, Q.; Qu, Y.; Xu, Y.; Wang, Y. Achieving highly efficient narrowband sky-blue electroluminescence with alleviated efficiency roll-off by molecular-structure regulation and device-configuration optimization. *J. Mater. Chem. C* **2022**, *10* (41), 15408–15415. DOI: 10.1039/d2tc03249j.

(2) Yang, M.; Park, I. S.; Yasuda, T. Full-Color, Narrowband, and High-Efficiency Electroluminescence from Boron and Carbazole Embedded Polycyclic Heteroaromatics. *J. Am. Chem. Soc.* **2020**, *142* (46), 19468–19472. DOI: 10.1021/jacs.0c10081.

(3) Gao, R. R.; Chen, C.; Huang, Y. B.; Wang, X. Y. Multi-Resonance 1,4-BN-Heteroarene for Filterless Narrowband Photodetector. *Angew. Chem. Int. Ed.* **2025**, *64* (10), e202500006. DOI: 10.1002/anie.202500006.

(4) Smallwood, I. M. *Handbook of Organic Solvent Properties*; Butterworth-Heinemann: Oxford, 1996.

(5) Frisch, M. J.; Trucks, G. W.; Schlegel, H. B.; Scuseria, G. E.; Robb, M. A.; Cheeseman, J. R.; Scalmani, G.; Barone, V.; Petersson, G. A.; Nakatsuji, H.; et al. *Gaussian 16*, rev. A.03; Gaussian, Inc.: Wallingford, CT, 2016.

(6) Becke, A. D. Density-functional thermochemistry. III. The role of exact exchange. *J. Chem. Phys.* **1993**, *98* (7), 5648–5652.

(7) Escudero, D.; Laurent, A. D.; Jacquemin, D. Time-dependent density functional theory: A tool to explore excited states. In *Handbook of computational chemistry*, Leszczynski, J. Ed.; Springer: Cham, 2017; pp 927–961.
